# Reduced expression of E-cadherin correlates with poor prognosis and unfavorable clinicopathological features in gastric carcinoma: a meta-analysis

**DOI:** 10.18632/aging.205929

**Published:** 2024-06-12

**Authors:** Genlin Lu, Zhai Cai, Renya Jiang, Fei Tong, Jinming Tu, Yandong Chen, Yinglan Fu, Jingyi Sun, Tao Zhang

**Affiliations:** 1 Department of General Surgery (Key Disciplines of Medicine in Quzhou City), Longyou County People’s Hospital, Longyou People’s Hospital Affiliated with Sir Run Run Shaw Hospital, Zhejiang University School of Medicine, Quzhou 324400, China; 2 Department of General Surgery, Zhujiang Hospital of Southern Medical University, Guangzhou 510280, China; 3 Department of Hepatobiliary Surgery, Quzhou People’s Hospital, Quzhou 324000, China; 4 Department of Gastroenterology, Longyou County People’s Hospital, Longyou People’s Hospital Affiliated with Sir Run Run Shaw Hospital, Zhejiang University School of Medicine, Quzhou 324400, China

**Keywords:** gastric carcinoma, E-cadherin, prognosis, clinicopathological feature, risk factors

## Abstract

Backgrounds: Gastric carcinoma (GC) is one of the most fatal human malignancies globally, with a median survival time less than 1 year. E-cadherin exerts a crucial role in the development and progression of GC as an adhesive, invasive suppressor gene. Whether reduced E-cadherin has an impact on prognosis, clinicopathological features for GC has been well studied, but no conclusive results has been obtained.

Methods: Eligible studies and relevant data were obtained from PubMed, Elsevier, Embase, Cochrane Library and Web of Science databases until June 30, 2023. A fixed- or random-effects model was used to calculate pooled odds ratios (OR) and 95% confidence intervals (CI). Correlation of E-cadherin expression with overall survival (OS), clinicopathological features and risk factors were evaluated.

Results: 36 studies fulfilled the selected criteria. 9048 cases were included. This meta-analysis showed that patients with GC with reduced E-cadherin had unfavourable clinicopathological features and poor OS. The pooled ORs of one-, three- and five-year OS were 0.38 (*n* = 25 studies, 95%CI: 0.25–0.57, Z = 4.61, *P* < 0.00001), 0.33 (*n* = 25 studies, 95% CI: 0.23–0.47, Z = 6.22, *P* < 0.00001), 0.27 (*n* = 22 studies, 95% CI: 0.18–0.41, Z = 6.23, *P* < 0.00001), respectively. Moreover, reduced E-cadherin expression significantly correlated with differentiation grade (OR = 0.29, 95% CI: 0.22–0.39, Z = 8.58, *P* < 0.00001), depth of invasion (OR = 0.49, 95% CI: 0.36–0.66, Z = 4.58, *P* < 0.00001), lymphatic node metastasis (OR = 0.49, 95% CI: 0.38–0.64, Z = 5.38, *P* < 0.00001), distant metastasis (OR = 2.24, 95% CI: 1.62–3.09, Z = 4.88, *P* < 0.00001), peritoneal metastasis (OR = 2.17, 95% CI: 1.39–3.39, Z = 3.40, *P* = 0.0007), TNM stage (OR = 0.41, 95% CI: 0.28–0.61, Z = 4.44, *P* < 0.00001), lymphatic vessel invasion (OR = 1.77, 95% CI: 1.11–2.82, Z = 2.39, *P* = 0.02), vascular invasion (OR = 1.55, 95% CI: 1.22–1.96, Z = 3.58, *P* = 0.0003), Lauren type (OR = 0.35, 95% CI: 0.21–0.57, Z = 4.14, *P* < 0.0001), Borrmann classification (OR = 0.50, 95% CI: 0.25–0.99, Z = 1.97, *P* = 0.048) and tumor size (≥5 cm vs. <5 cm: OR = 1.73, 95% CI: 1.34–2.23, Z = 4.19, *P* < 0.0001; ≥6 cm vs. <6 cm: OR = 2.29, 95% CI: 1.51–3.49, Z = 3.87, *P* = 0.0001). No significant association was observed between reduced E-cadherin expression and liver metastasis, perineural invasion, alcohol consumption, smoking status, familial history, Helicobacter pylori (HP) infection.

Conclusions: The reduced expression of E-cadherin is significantly correlated with poor OS and unfavourable clinicopathological features in GC. The expression level of E-cadherin not only serves as a predictor for disease progression and prognosis in GC but also emerges as a novel therapeutic target.

## INTRODUCTION

Gastric carcinoma (GC) is one of the most fatal human malignancies globally [1]. It was reported that 1 million new patients suffer from GC annually [1]. It was estimated that 784000 deaths were caused by GC globally in 2018 [1]. Endoscopic mucosal resection or endoscopic submucosal dissection is adopted for patients with early GC. Gastrectomy with D_2_ lymphadenectomy is suitable for locally advanced GC. A comprehensive plan including chemotherapy, immunotherapy, anti-angiogenic therapy, and trastuzumab for Her2-positive GC, improves overall survival (OS). Nonetheless median OS is within 12 months. It is believed that Helicobacter pylori (HP) infection, dinking, hereditary tendency, salted and smoked food intake, and gastroesophageal reflux disease are risk factors for GC [2]. There is an urgent need to understand genes involved in the initiation, progression, and prognosis of gastric cancer, which exhibits a high level of heterogeneity both at the molecular and phenotypic levels.

E-cadherin (E-cad) is a member of Ca^2+^-dependent membrane glycoprotein, encoded by CDH1 gene which is crucial for preserving epithelial cell-cell junctions and cell polarity, and suppresses tumor growth, metastasis and invasion in numerous cancers comprising GC. E-cadherin exerts its effects on the Wnt-signaling pathway by negatively regulating the quantity of unbound β-catenin, which is indispensable in the pathogenesis of GC [3, 4]. Low E-cadherin expression in GC is attributed to mutation in the CDH1 gene on chromosome 16q22.1 [5], E-cadherin promoter hypermethylation [6], and transcriptional repression resulting from Snail [7] and Sip-1 [8] binding to the CDH1-E box.

As far as the correlations between E-cad expression and clinical characteristics, as well as prognoses for patients with GC are concerned, vast amounts of work have been done but study results exhibit great diversity and inconsistency. Furthermore, the quantity of participants recruited for each research is not sufficiently large. So, this article was conducted to systematically and comprehensively evaluate its correlations.

## MATERIALS AND METHODS

### Data retrieval

The articles published before June 30, 2023 in the PubMed, Elsevier, Embase, Cochrane Library, and Web of Science databases were systematically searched. The terms used in the search were as follows: “E-Cadherin”, “prognosis”, and “stomach neoplasms”. The reference lists of publications were retrieved by manual. Only English-language studies were encompassed in the selection process.

### Criteria for inclusion and exclusion

Inclusion criteria: (1) Pathological diagnosis is GC; (2) Data about E-cadherin expression, OS, and clinical characteristics were comprehensive; (3) E-cadherin expression was detected by immunohistochemical staining, western blotting, immunofluorescence; (4) When multiple studies were published by a single author, only the one with the highest quality was included; (5) Study written in English was enrolled.

Exclusion criteria: (1) Abstracts, reviews, editorials, case reports, as well as letters; (2) Study subjects are cell lines, and animals; (3) Overlapping publication; (4) Information about E-cadherin expression, OS, as well as clinical characteristics was unavailable.

### Data retrieval and compilation and evaluation of literature quality

Each study was evaluated and relevant characteristics were extracted by three reviewers (GLL, JYS and RYJ) independently. The data were presented as follows: (1) authors and publication time; (2) clinical characteristics; (3) level of evidence, (4) the rate of E-cadherin expression, (5) OS data (Table 1). Literature quality was evaluated by Newcastle-Ottawa scale (NOS) [9].

**Table 1:** Characteristics of studies included in the meta-analysis.

**First author and year**	**Country or region**	**Mean age**	**Gender (M/F)**	**Level of evidence**	**Stage**	**Clinicopathological features**	**Method**	**Provided- OS data**	**No. of patients**	**Reduced/total E-cadherin (%)**
Bahnassy [11] 2018	Egypt	53.2 ± 14.1	126/66	5	NR	NR	IHC	NR	192	84/192 (43.8)
Saad [12] 2010	Egypt	NR	16/14	4	I–IV	D, T	IHC	Yes	30	11/30 (36.7)
Ayed-Guerfali [13] 2014	Tunisian	55	45/35	3	I–IV	D, T, M	IHC	Yes	80	47/80 (58.8)
Cai [14] 2001	China	63 (37–82)	56/79	4	I–II	D	IHC	NR	135	77/135 (57.0)
Chen [15] 2003	China Twain	46 (27-64)	NR	4	I–IV	D, M	IHC	Yes	84	29/84 (34.5)
Czyzewska [16] 2010	Poland	NR	69/29	4	NR	T	IHC	Yes	91	37/91 (40.7)
Dong [17] 2014	China	60 (35–81)	106/22	4	I–III	D, T, M	IHC	Yes	128	73/128 (57.0)
Gabbert [18] 1996	Germany	64.9 (23–90)	255/158	4	I–IV	D, T	IHC	Yes	413	124/413 (30.0)
Guo [19] 2019	China	62 (40–83)	45/24	5	I–IV	D	IHC	Yes	69	44/69 (63.8)
Guo [20] 2014	China	61 (37–83)	121/38	4	I–IV	D, T	IHC	Yes	159	113/159 (71.1)
Hu [21] 2013	China	55 (30–73)	145/44	3	NR	D, T	IHC	NR	189	148/189 (78.3)
Hu [22] 2023	China	38–78	48/17	4	I–III	D, T	IHC	Yes	65	29/65 (44.6)
Jawhari [23] 1997	UK	70 (33–84)	62/27	3	NR	D	IHC	Yes	89	21/89 (23.6)
Joo [24] 2000	Korea	55.2 ± 10.3	38/27	4	I–IV	D, T, M	IHC	Yes	65	34/65 (52.3)
Joo [25] 2001	Korea	NR	70/44	3	I–IV	D, M	IHC	Yes	114	40/114 (35.1)
Yi Kim [26] 2007	Korea	58.7 (37–83)	38/22	3	I–IV	D, M	IHC	NR	60	33/60 (55)
Kim [27] 2009	Korea	54.8	396/168	3	NR	NR	IHC	Yes	564	240/564 (42.6)
LAZĂR [28] 2008	Rumania Europe	59.3 (30–78)	43/18	3	I–IV	D, T, M	IHC	Yes	61	31/61 (50.8)
Li [29] 2012	China	55 (25–80)	72/42	3	I–IV	D, T, M	IHC	Yes	114	69/114 (60.5)
Li [30] 2015	China	55 (28–78)	51/18	4	I–III	D, T	IHC	Yes	69	27/69 (39.1)
Mohamed [31] 2019	Egypt	53 ± 14	42/22	5	NR	D	IHC	NR	64	28/64 (43.8)
Ramesh [32] 1999	UK	68 (57–87)	31/9	3	NR	D	IHC	NR	40	30/40 (75.0)
Shino [33] 1995	Japan	62 (24–83)	77/44	4	NR	D, M	IHC	NR	121	39/121 (32.2)
Song [34] 2004	Korea	55.8 ± 11.6	65/30	3	I–II	D	IHC	NR	95	34/95 (35.8)
Sun [35] 2019	China	62 (29–79)	34/21	4	I–IV	D, T	IHC	Yes	55	22/55 (40.0)
Uchikado [36] 2011	Japan	65 (22–88)	113/51	4	I–IV	D, T, M	IHC	NR	164	92/164 (56.1)
Wang [37] 2022	China	NR	3607/954	4	I–IV	D, T	IHC	Yes	4561	725/4561 (15.9)
Xu [38] 2019	China	59.58 (18–94)	71/37	4	I–IV	D, T, M	IHC	NR	108	44/108 (40.7)
Xu [39] 2016	China	57.8 ± 10.3	76/29	4	I–IV	D, T	IHC	NR	105	57/105 (52.4)
Yonemura [40] 1995	Japan	63.4 (27–86)	NR	4	I–IV	D, T	IHC	Yes	125	83/125 (66.4)
Yonemura [41] 1997	Japan	NR	NR	4	I–IV	D, T, M	IHC	Yes	127	84/127 (66.1)
Yonemura [42] 2000	Japan	NR	NR	3	NR	D, T, M	IHC	Yes	92	66/92 (71.7)
Zhong [43] 2008	China	59 (33–82)	87/31	3	I–IV	D, M	IHC	Yes	118	83/118 (70.3)
Zhou [44] 2002	China	54.5 (22–77)	123/40	5	NR	D, T	IHC	Yes	163	75/163 (46.0)
Zhou [45] 2010	China	54 (30–73)	153/47	3	NR	D, T	IHC	Yes	200	156/200 (78.0)
Zhou [46] 2016	China	33.8 ± 5.47	52/87	3	I–IV	NR	Western blot	Yes	139	79/139 (56.8)

Abbreviations: IHC: immunohistochemistry test; D: differentiation grade; T: depth of invasion; M: distant metastasis; OS: overall survival; NR: not reported.

### Statistical analysis

The Review Manager software (version 5.3) and Stata software (version 18) were utilized to generate pooled odds ratios (ORs) along with 95% confidence intervals (CIs) [10]. The associations between E-cad expression and overall survival (OS), clinicopathological features, and risk factors were evaluated. Stratification based on study origin was conducted through subgroup analysis and meta-regression [9, 10]. Funnel plots and Egger’s test were employed to evaluate publication bias. As the I² value exceeds 50%, there is considered to be significant heterogeneity. When the *P*-value is less than 0.05, it is deemed that statistical significance exists.

## RESULTS

### Selection of trials

**Figure 1:**
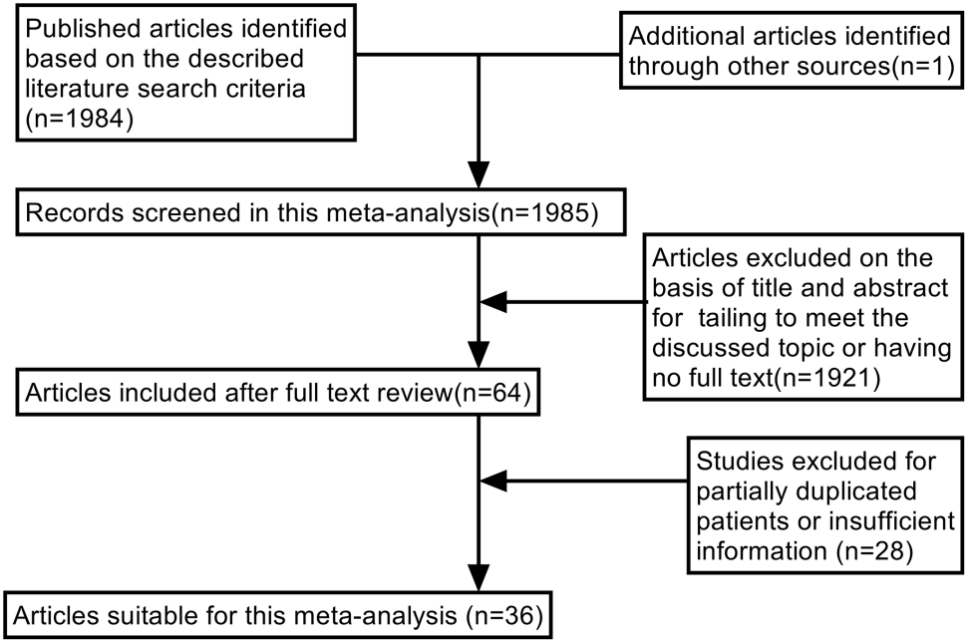
Flow chart of literature search strategies.

Figure 1 demonstrates that 36 studies met the inclusion criteria and were enrolled for analysis of the prognostic value of E-cad expression, as well as its association with clinical characteristics and risk factors for GC (of the 1985 publications, 1921 studies were excluded due to incomplete content, 16 were excluded because they lacked sufficient data to calculate OS, and 12 were excluded as their data overlapped with those of other studies).

### Study characteristics

Table 1 shows the data on E-cad expression, OS, clinical features, and risk factors from 36 enrolled studies eligible for the meta-analysis. A total of 9048 patients with GC were included, among whom 2998 patients exhibited lower levels of E-cad expression. The expression of E-cadherin in each study was determined by immunohistochemical staining, western blotting, immunofluorescence, or other methods, all conducted without subjective interference.

### Quality assessment

Table 1 indicates that 4 studies scored 5 points [11, 20, 31, 44], 18studies scored 4 points [12, 14–19, 22, 24, 30, 33, 35–41], 14 studies scored 3 points [13, 21, 23, 25–29, 32, 34, 42, 43, 45, 46]. When the score of NOS is over 5 points, the studies is highly qualified.

### Impact of E-cadherin expression on OS

**Figure 2:**
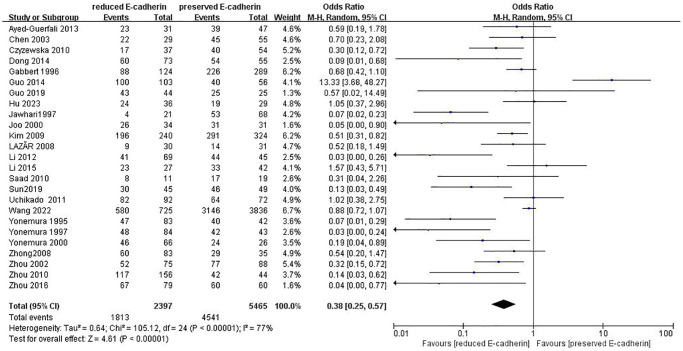
Forest plot of the odds ratio for the correlation of E-cadherin expression with one-year overall survival.

**Figure 3:**
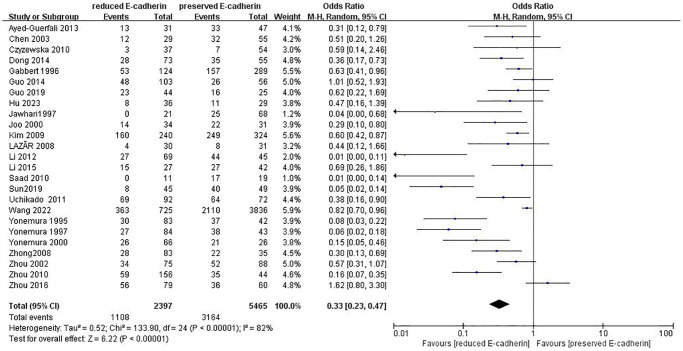
Forest plot of the odds ratio for the correlation of E-cadherin expression with three-year overall survival.

**Figure 4:**
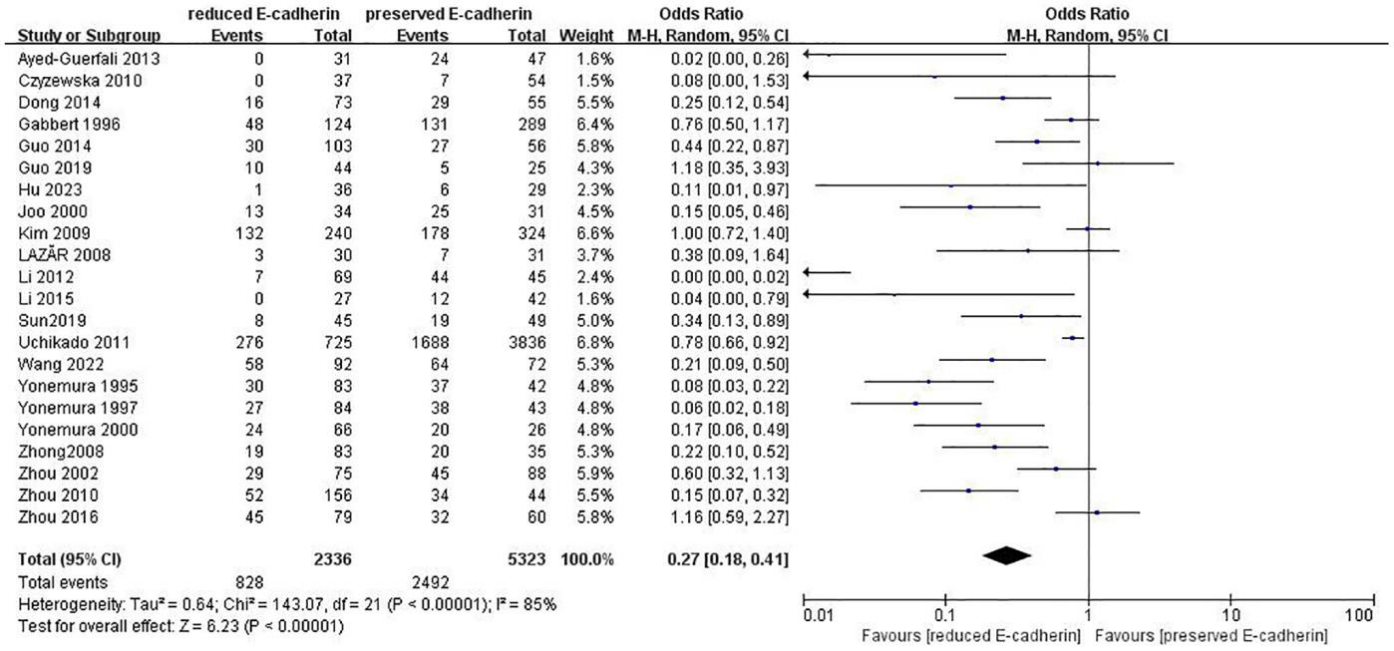
Forest plot of the odds ratio for the correlation of E-cadherin expression with five-year overall survival.

As indicated in Figures 2–4 and Table 2, there are predominant correlations between reduced E-cadherin and poor one-, three-, and five-year OS, respectively (*n* = 25 studies [12, 13, 15–20, 22–24, 27–30, 35–37, 40–46], OR: 0.38, 95% CI: 0.25–0.57, Z = 4.61, *P* < 0.00001; *n* = 25 studies [12, 13, 15–20, 22–24, 27–30, 35–37, 40–46], OR: 0.33, 95% CI: 0.23–0.47, Z = 6.22, *P* < 0.00001; *n* = 22 studies [13, 16–20, 22, 24, 27–30, 35–37, 40–46], OR: 0.27, 95% CI: 0.18–0.41, Z = 6.23, *P* < 0.00001, respectively). The I^2^ statistic of the one-, three-, five-year OS was 77%, 82%, 85% respectively. The results of subgroup analyses revealed that reduced E-cadherin was predominantly associated with three-, five-year OS of patients with GC in China, Japan and Korea, as well as one-year OS of patients with GC in Japan, as illustrated in Table 3. It was concluded that reduced E-cad had a worse impact on prognosis in GC.

**Table 2:** Correlation between E-cadherin expression and OS, clinicopathological feature, and risk factors for GC.

**Outcome of interest**	**Number of studies**	**Number of tissue samples**	**OR (95% CI)**	***Z*-value**	***P*-value**	**I^2^(%)**
One-year overall survival	25	RE = 2397, PE = 5465	0.38 (0.25–0.57)	4.61	<0.00001	77
Three-year overall survival	25	RE = 2397, PE = 5465	0.33 (0.23–0.47)	6.22	<0.00001	82
Five-year overall survival	22	RE = 2336, PE = 5323	0.27 (0.18–0.41)	6.23	<0.00001	85
Depth of invasion	22	RE = 2155, PE = 5046	0.49 (0.36–0.66)	4.58	<0.00001	65
Lymphatic node metastasis	32	RE = 2700, PE = 5536	0.49 (0.38–0.64)	5.38	<0.00001	73
Distant metastasis	13	RE = 662, PE = 621	2.24 (1.62–3.09)	4.88	<0.00001	34
Lauren type	19	RE = 1139, PE = 1189	0.35 (0.21–0.57)	4.14	<0.0001	84
Differentiation grade	32	RE = 2519, PE = 5497	0.29 (0.22–0.39)	8.58	<0.00001	74
TNM stage	23	RE = 1984, PE = 5068	0.41 (0.28–0.61)	4.44	<0.00001	79
Lymphatic vessel invasion	9	RE = 601, PE = 679	1.77 (1.11–2.82)	2.39	0.02	62
Vascular invasion	13	RE = 829, PE = 850	1.55 (1.22–1.96)	3.58	0.0003	17
Peritoneal metastasis	6	RE = 358, PE = 338	2.17 (1.39–3.39)	3.40	0.0007	36
Tumor size (≥5 cm vs. <5 cm)	10	RE = 729, PE = 488	1.73 (1.34–2.23)	4.19	<0.0001	10
Tumor size (≥6cm vs. <6 cm)	3	RE = 270, PE = 141	2.29 (1.51–3.49)	3.87	0.0001	4
Borrmann classification	6	RE = 397, PE = 327	0.5 (0.25–0.99)	1.97	0.048	56
Liver metastasis	5	RE = 320, PE = 246	1.21 (0.67–2.18)	0.62	0.53	48
Perineural invasion	3	RE = 230, PE = 176	1.03 (0.46–2.30)	0.06	0.95	65
Hp infection	4	RE = 244, PE = 222	0.65 (0.29–1.46)	1.04	0.3	75
Smoking status	2	RE = 405, PE = 2022	1.1 (0.94–1.28)	1.14	0.25	0
Alcohol consumption	2	RE = 758, PE = 3897	1 (0.85–1.19)	0.03	0.98	0
Familial history	2	RE = 804, PE = 3896	0.93 (0.78–1.12)	0.74	0.46	37

Abbreviations: Hp: Helicobacter pylori, RE: reduced E-cadherin expression, PE: preserved E-cadherin expression; OR: odds ratio; CI: confidence interval; TNM stage: depth of tumor invasion, lymphatic node metastasis, distant metastasis stage classification.

### Association between E-cadherin expression and clinical characteristics

**Figure 5:**
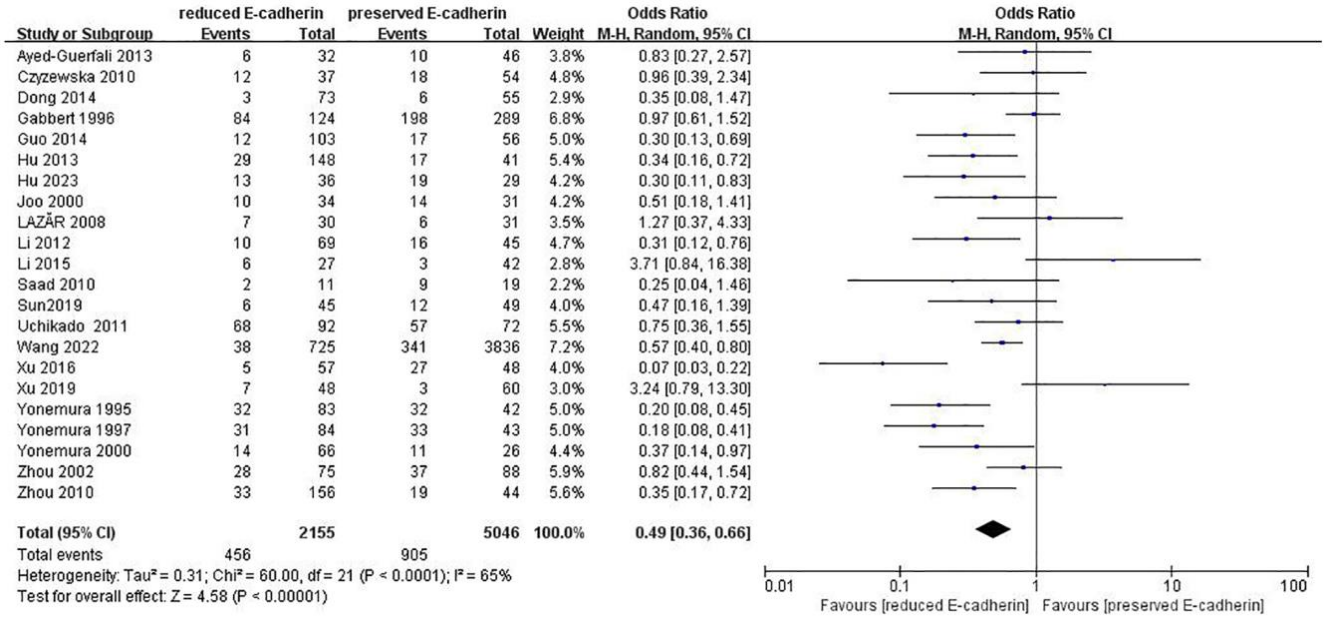
Forest plot of the odds ratio for the correlation of E-cadherin expression with depth of invasion.

**Figure 6:**
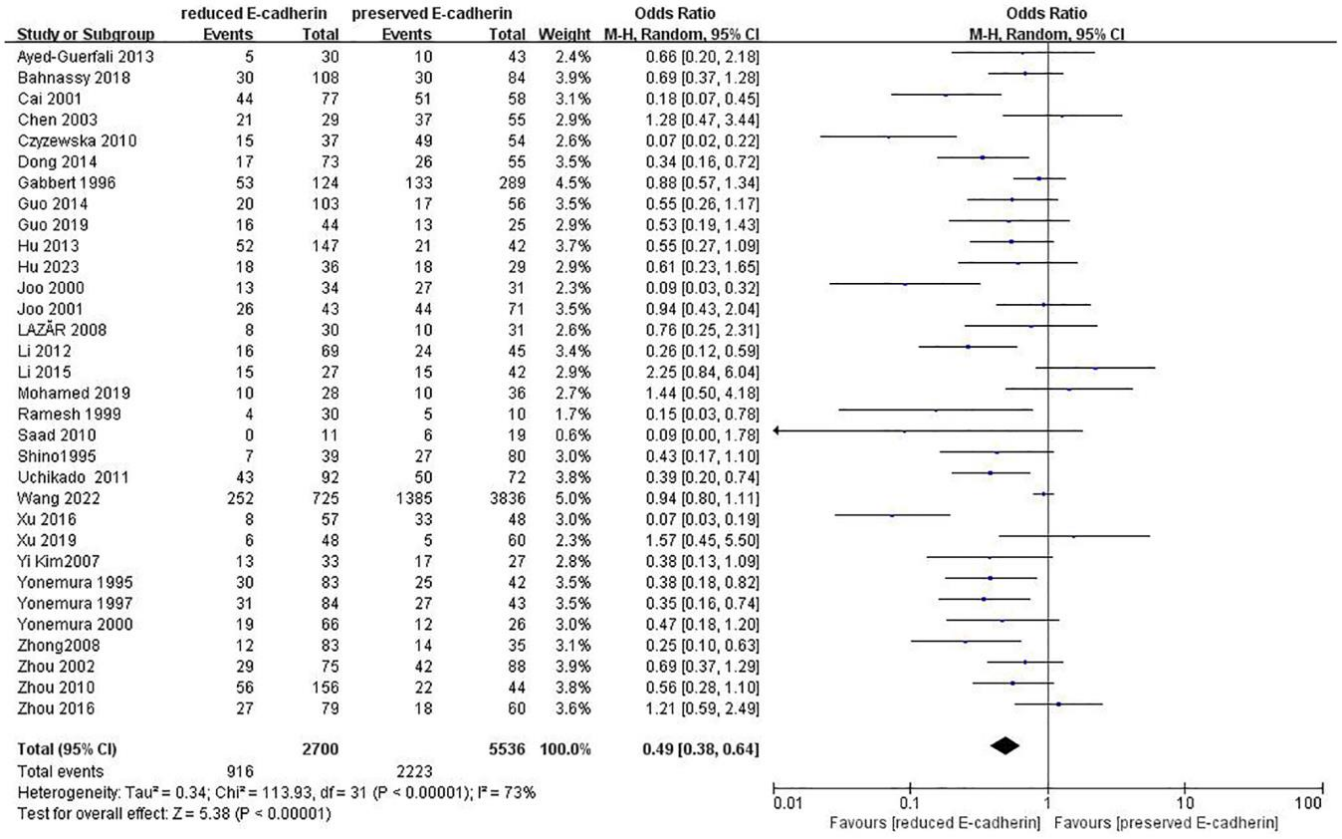
Forest plot of the odds ratio for the correlation of E-cadherin expression with lymphatic node metastasis.

**Figure 7:**
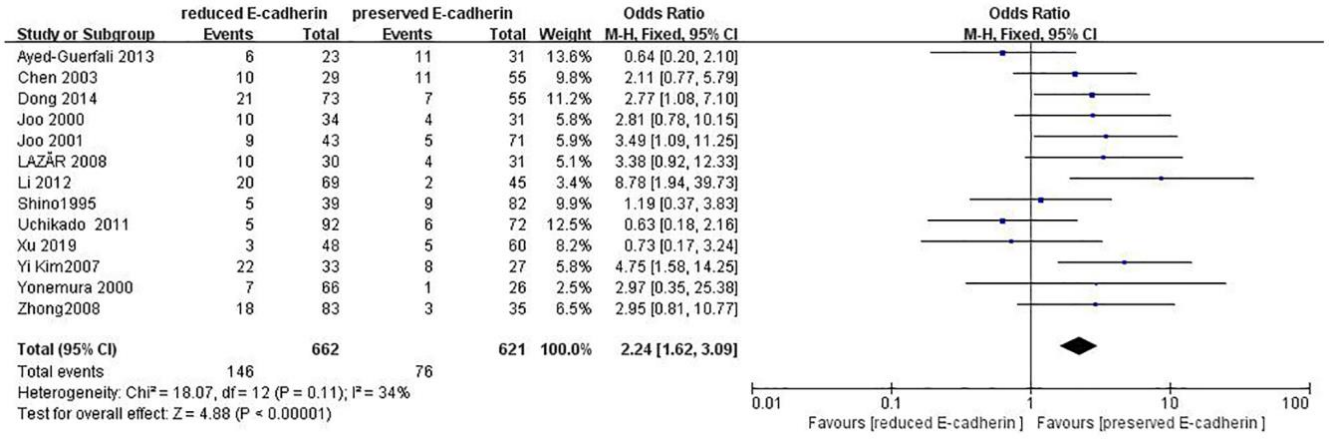
Forest plot of the odds ratio for the correlation of E-cadherin expression with distant metastasis.

**Figure 8:**
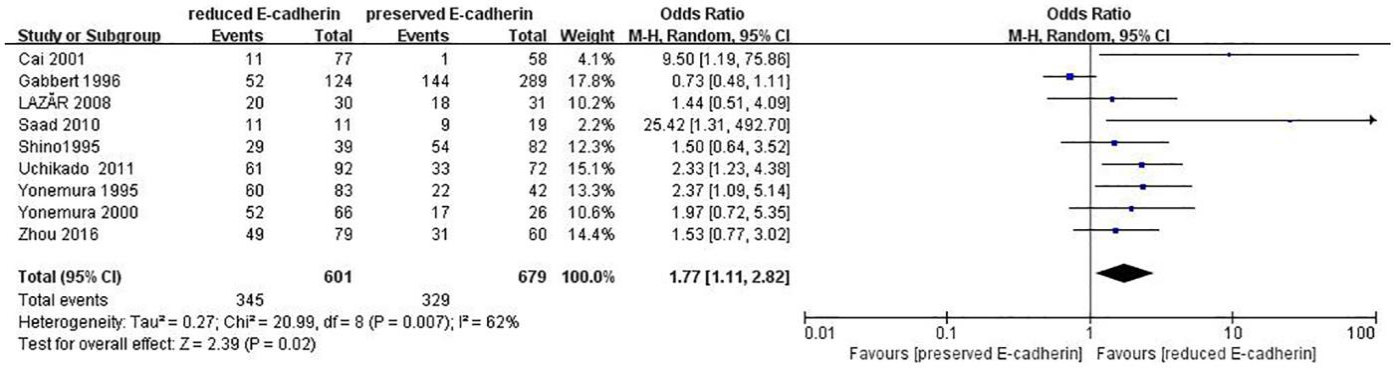
Forest plot of the odds ratio for the correlation of E-cadherin expression with lymphatic vessel invasion.

**Figure 9:**
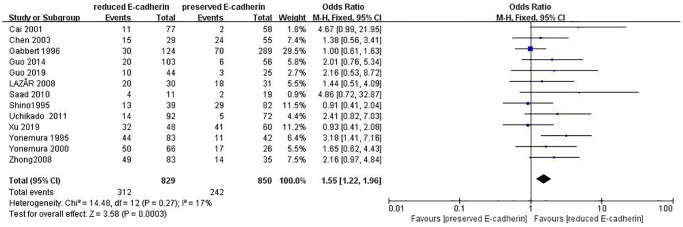
Forest plot of the odds ratio for the correlation of E-cadherin expression with vascular invasion.

**Figure 10:**
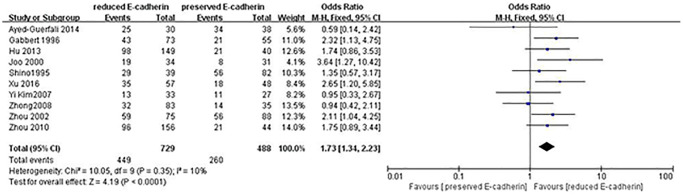
Forest plot of the odds ratio for the correlation of E-cadherin expression with tumor size (≥5 cm vs. <5 cm).

**Figure 11:**
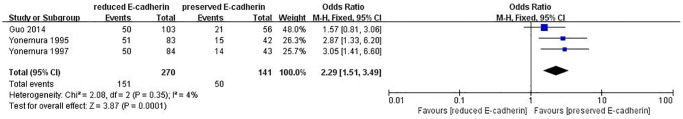
Forest plot of the odds ratio for the correlation of E-cadherin expression with tumor size (≥6 cm vs. <6 cm).

**Figure 12:**
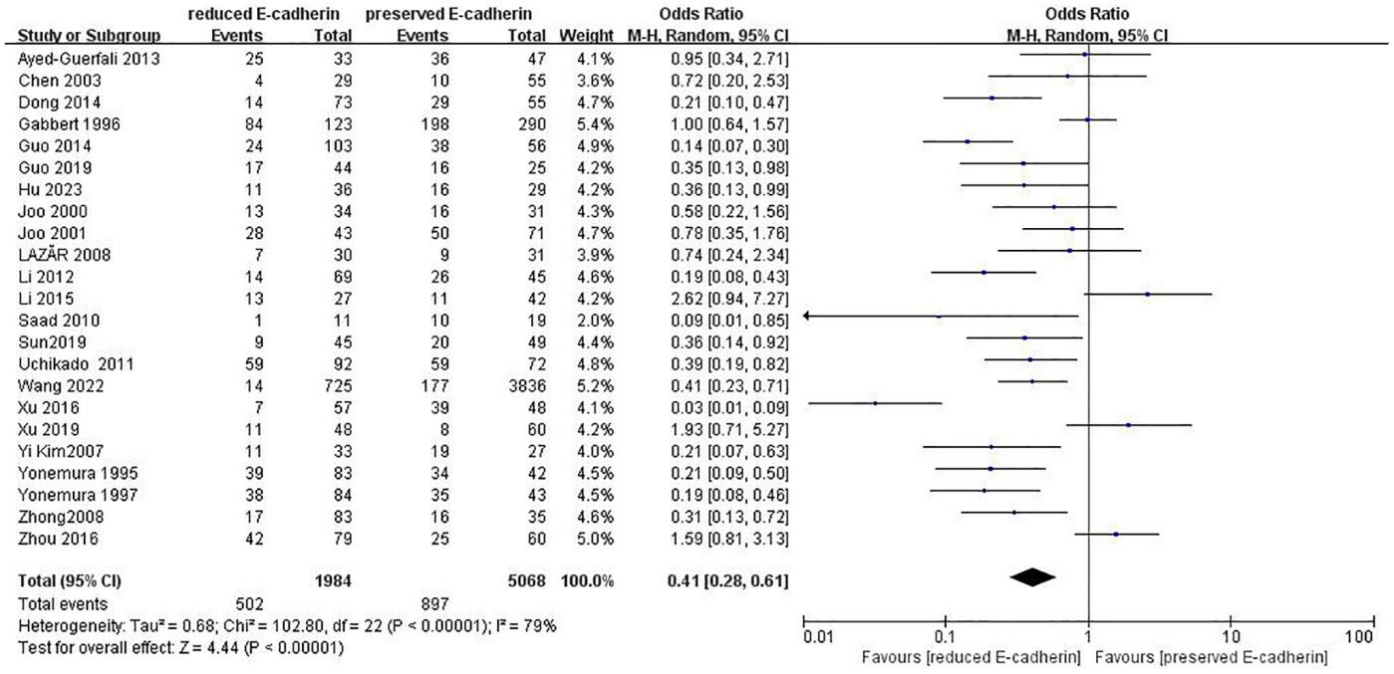
Forest plot of the odds ratio for the correlation of E-cadherin expression with TNM stage.

**Figure 13:**
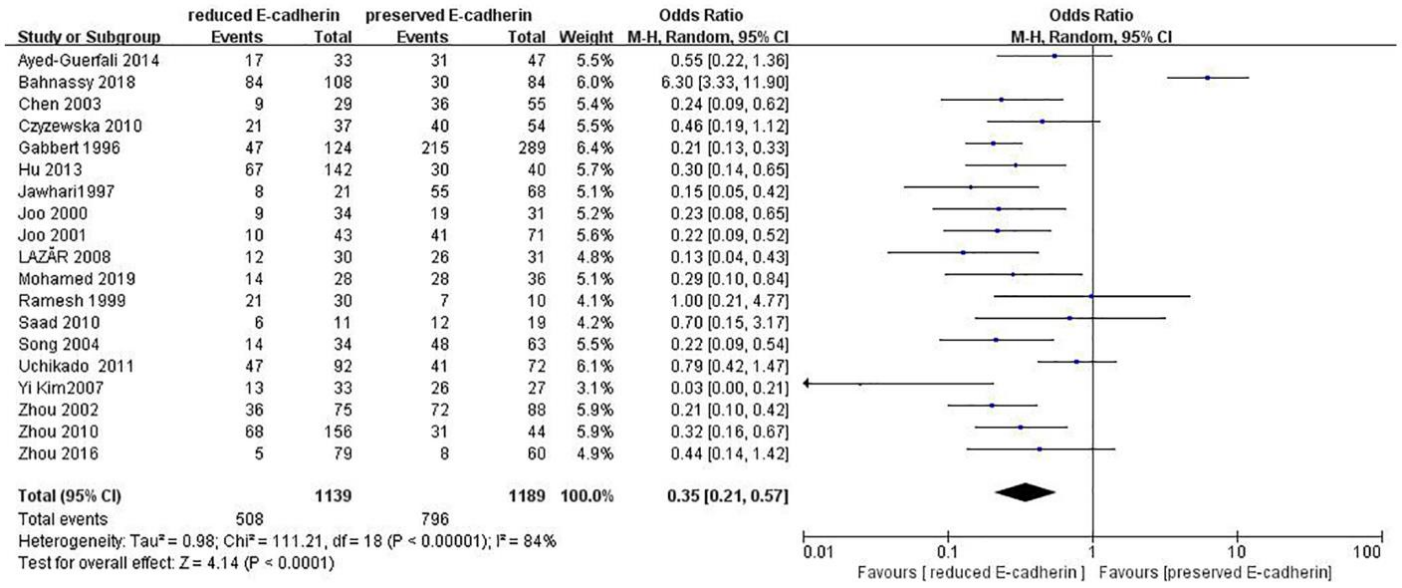
Forest plot of the odds ratio for the correlation of E-cadherin expression with Lauren type.

**Figure 14:**
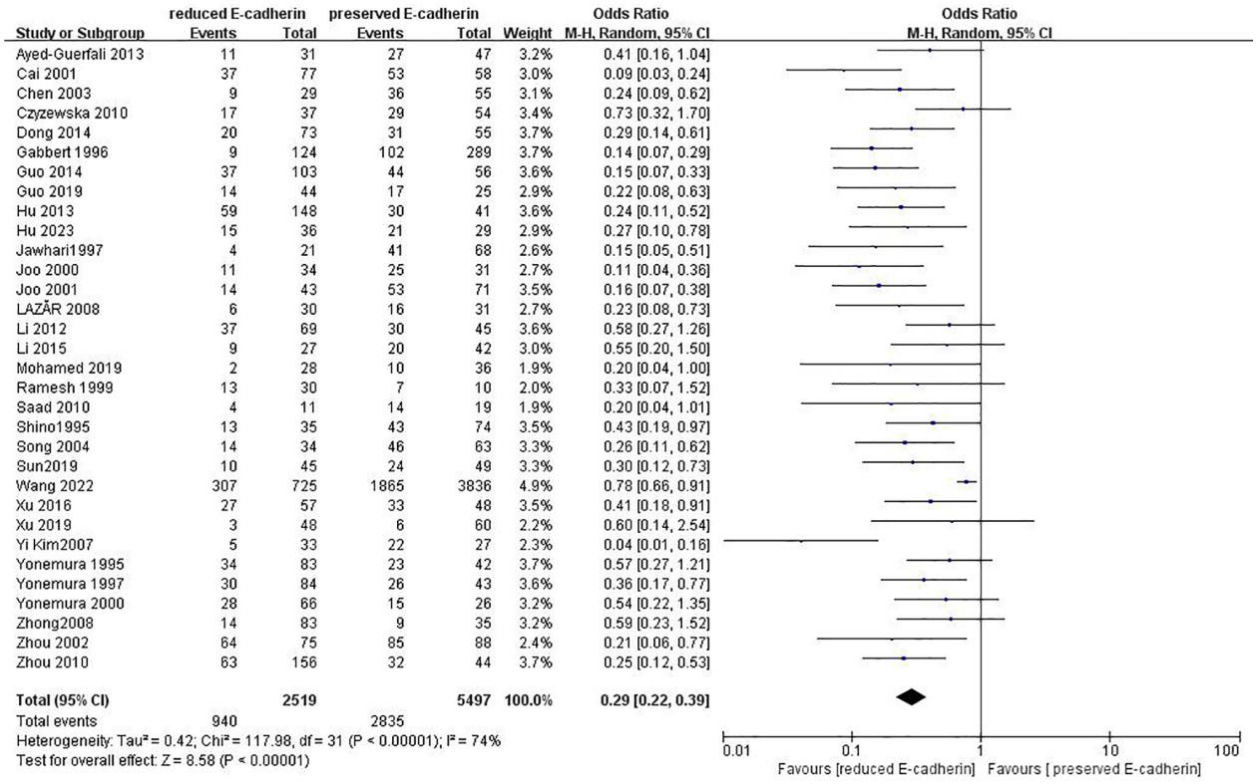
Forest plot of the odds ratio for the correlation of E-cadherin expression with differentiation grade.

**Figure 15:**
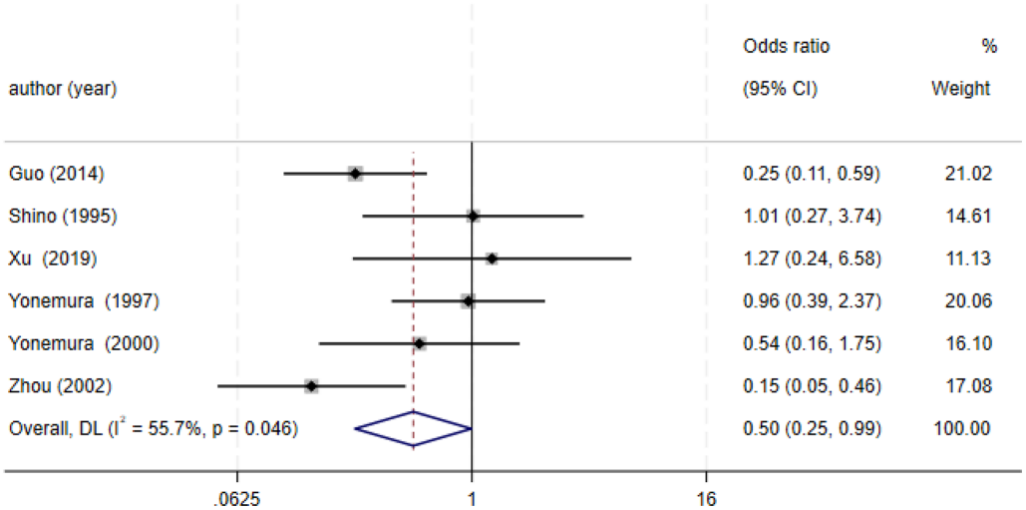
Forest plot of the odds ratio for the correlation of E-cadherin expression with Borrmann classification.

**Figure 16:**
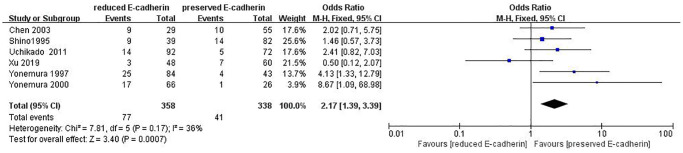
Forest plot of the odds ratio for the correlation of E-cadherin expression with peritoneal metastasis.

The correlations between E-cadherin expression and depth of invasion, differentiation grade, lymphatic node metastasis, distant metastasis, liver metastasis, peritoneal metastasis, TNM stage, perineural invasion, lymphatic vessel invasion, vascular invasion, Lauren type, Borrmann classification and tumor size were examined. 22 studies [12, 13, 16–19, 21–22, 24, 28–30, 35–42, 44, 45] assessed the association between E-cadherin expression and depth of invasion (T_1_+T_2_ vs. T_3_+T_4_) (OR: 0.49, 95% CI: 0.36–0.66, Z = 4.58, *P* < 0.00001, Figure 5). 32 studies [11, 12–22, 24–26, 28–33, 36–46] evaluated the correlation between E-cad expression and lymphatic node metastasis (negative vs. positive) (OR: 0.49, 95% CI: 0.38–0.64, Z = 5.38, *P* < 0.00001, Figure 6). The result of subgroup analysis displayed that reduced E-cad strikingly related to lymphatic node metastasis of patients with GC in China, Korea, Japan and other countries, as depicted in Table 3. 13 studies [13, 15, 17, 24–26, 28–29, 33, 36, 38, 42, 43] measured the correlation of E-cad expression with distant metastasis (Figure 7). The pooled OR was 2.24 (95% CI: 1.62–3.09, Z = 4.88, *P* < 0.00001). 9 studies [12, 14, 18, 28, 33, 36, 40, 42, 46] surveyed the correlation between E-cadherin expression and lymphatic vessel invasion (positive vs. negative) (OR: 1.77, 95% CI: 1.11–2.82, Z = 2.39, *P = 0.02,* Figure 8).13 studies [12, 14–15, 18–20, 28, 33, 36, 38, 40, 42, 43] analyzed the association between E-cadherin expression and vascular invasion (positive vs. negative) (OR: 1.55, 95% CI: 1.22–1.96, Z = 3.58, *P* = 0.0003, Figure 9). 10 studies [13, 18, 21, 24, 26, 33, 39, 43–45] evaluated the correlation of E-cad expression with tumor size (≥5 cm vs. <5 cm) (OR: 1.73, 95% CI: 1.34–2.23, Z = 4.19, *P* < 0.0001, Figure 10). 3 studies [20, 40, 41] evaluated the correlation between E-cadherin expression and tumor size (≥6 cm vs. <6 cm) (Figure 11). The pooled OR was 2.29 (95% CI: 1.51–3.49, Z = 3.87, *P* = 0.0001). 23 studies [12, 13, 15, 17–20, 22, 24–26, 28–30, 35–41, 43, 46] appraised the association of E-cadherin expression with TNM stage (I+II vs. III+IV) (OR:0.41,95% CI: 0.28-0.61, Z = 4.44, *P* < 0.00001, Figure 12). 19 studies [11–13, 15–16, 18, 21, 23–26, 28, 31–32, 34, 36, 44–46] estimated the association of E-cad expression with Lauren type (intestine-type vs. diffuse-type) (OR: 0.35, 95% CI: 0.21–0.57, Z = 4.14, *P* < 0.0001, Figure 13). 32 studies [12–26, 28–33, 35–46] examined the association between E-cadherin expression and differentiation grade (well or moderate-differentiated vs. poor- differentiated) (OR: 0.29, 95% CI: 0.22–0.39, Z = 8.58, *P* < 0.00001, Figure 14). 6 studies [19, 33, 38, 41, 42, 44] detected the association of E-cad expression with Borrmann classification (Borrmann I+II vs. Borrmann III+IV) (OR: 0.50, 95% CI: 0.25–0.99, Z = 1.97, *P* = 0.048, Figure 15). 6 studies [15, 33, 36, 38, 41, 42] investigated the association of E-cad expression and peritoneal metastasis (OR: 2.17, 95% CI: 1.39–3.39, Z = 3.40, *P* = 0.0007, Figure 16). As shown in Supplementary Figures 1 and 2, There is no significant association of E-cadherin expression with liver metastasis or perineural invasion. Taken together, these results above demonstrate that reduced E-cadherin is predominantly correlated with unfavourable clinicopathological parameters.

**Table 3:** Subgroup analysis for E-cadherin expression with OS and lymphatic node metastasis in GC.

**Factors**	**Subgroup**	**Number of tissue samples**	**Number of studies**	***Z*-value**	**OR (95% CI)**	***P*-value**	**I^2^ (%)**	***P*-value (Egger’s test)**
One-year overall survival								
	China	RE = 1544, PE = 4419	11	1.92	0.51(0.26–1.01)	0.06	77	0.235
	Japan	RE = 325, PE = 183	4	2.13	0.16 (0.03–0.86)	0.03	82	0.200
	Korea	RE = 274, PE = 355	4	1.71	0.27 (0.06–1.21)	0.09	77	0.059
	Other countries	RE = 254, PE = 508	6	2.98	0.36 (0.19–0.71)	0.02	65	0.489
Three-year overall survival								
	China	RE = 1544, PE = 4419	11	3.44	0.45 (0.29–0.71)	0.00006	81	0.063
	Japan	RE = 325, PE = 183	4	4.65	0.13 (0.06–0.31)	<0.00001	65	0.52
	Korea	RE = 274, PE = 355	4	2.25	0.29 (0.10–0.86)	0.02	81	0.218
	Other countries	RE = 254, PE = 508	6	2.71	0.31 (0.13–0.72)	0.0007	63	0.233
Five-year overall survival								
	China	RE = 1515, PE = 4364	10	3.59	0.44 (0.28–0.69)	<0.0001	78	0.052
	Japan	RE = 325, PE = 183	4	6.92	0.12 (0.07–0.22)	<0.0001	33	0.064
	Korea	RE = 274, PE = 355	4	2.14	0.08 (0.01–0.81)	0.033	82	0.272
	Other countries	RE = 222, PE = 421	4	1.94	0.24 (0.06–1.01)	0.052	69	0.079
Lymphatic node metastasis								
	China	RE = 1828, PE = 4578	16	3.26	0.54 (0.38–0.78)	0.001	77	0.829
	Japan	RE = 364, PE = 263	5	5.20	0.39 (0.28–0.56)	<0.0001	0	0.627
	Korea	RE = 110, PE = 129	3	2.04	0.44 (0.33–0.59)	0.042	30	0.92
	Other countries	RE = 398, PE = 566	8	2.20	0.49 (0.26–0.93)	0.028	71	0.064

Abbreviations: OR: odds ratio; CI: confidence interval.

### Correlation of E-cadherin expression with risk factors

The associations of E-cadherin expression with risk factors, including alcohol consumption, smoking status, familial history, and HP infection were evaluated. As depicted in Table 2 and Supplementary Figures 3–6, E-cadherin expression is not correlated with alcohol consumption, smoking status, familial history and HP infection.

### Publication bias

Egger’s test manifests that there is not any publication bias for studies included in analysis of OS, risk factors, and clinicopathological parameters except differentiation grade (*p* = 0.0001). As shown in Supplementary Figures 7–26, the funnel plots for publication bias were symmetric except for some degree of asymmetry of studies involved in the analysis of differentiation grade (Supplementary Figure 27).

## DISCUSSION

A personalized treatment plan, including surgery, chemotherapy, anti-angiogenic therapy, and immunotherapy, trastuzumab for Her2- positive GC, can help patients with GC improve their OS.

However, the median survival is within 12 months. It is demonstrated that E-cad is crucial for tumor development, invasion, metastasis in GC. There is no consensus about impact of E-cadherin expression on prognosis and clinical characteristics of patients with GC. In this meta-analysis 9048 cases from 36 eligible studies were analyzed to elucidate its correlation.

OR is a measure of effect size commonly used in meta-analysis, particularly when dealing with dichotomous outcomes, which is also a statistic that quantifies the strength of outcome between the correlation of an exposure with an outcome. A pooled OR, is a single and overall estimate of the effect, which is obtained in a meta-analysis to combine the results from multiple studies. The resulting pooled OR provides a more precise and reliable estimate of the effect than any single study alone.

Recent researches have disclosed that decreased E-cadherin expression in GC ranges from 15.9% [37] to 85.4% [3] by IHC tests. This study denoted that the lower levels of E-cad in GC occur at the rate of 33.1%. Zhou et al., revealed that a normal state of E-cadherin expression is essential for the favourable prognosis of patients with GC [46]. As demonstrated in this article, reduced expression of E-cadherin was significantly correlated with one-, three-, and five-year overall survival (OS) of patients with gastric cancer, especially in China, Korea, and Japan. No publication bias was observed in the subgroup analysis conducted in each of these regions. It is consistent with the result of Zhou et al.

Regarding clinicopathological parameters, this study found that lower levels of E-cad expression are predominantly correlated with deeper invasion, poor differentiation, higher TNM staging, distant metastasis, lymphatic node metastasis, peritoneal metastasis, vascular invasion, lymphatic vessel invasion, greater tumor size, diffuse type of Lauren classification, and Borrmann III+IV. No obvious association exists between lower E-cadherin level and liver metastasis and perineural invasion. A normal state of E-cadherin expression is key to favourable clinicopathological characteristics of GC.

The E-cadherin–catenin complex consists of E-cadherin, p120, β-catenin, and α-catenin, and inhibits individual cell motility. CDH1 gene mutation, including methylation, leads to reduced E-cadherin protein expression, thereby triggering epithelial-mesenchymal transition and resulting in the loss of cell adhesion capacity [13, 17, 23–25]. The E-Cadherin/Wnt/ β-catenin pathway [3, 47] and the E-Cadherin/EGFR/ RAS/RAF/MEK pathway [48] impact on patients’ prognosis in GC, as described below. The reduction of E-cadherin expression upregulates the Wnt/β-catenin pathway and increases the expression of c-Myc, cyclins, and specific MMPs (e.g., MMP-3), and represses the expression of PTEN, which promotes cell proliferation and oncogenesis [3, 48, 49]. Upregulation of transcription factors including Snail, Twist, and Zeb-1 causes reduced E-cadherin expression, which promotes cell motility [7, 27, 36].

It is believed that Helicobacter pylori (HP) infection, dinking, hereditary tendency, salted and smoked food intake, and gastroesophageal reflux disease are risk factors for GC [2]. Worldwide incidence of distal GC related to HP seems to be on the rise. HP silences E-cad gene by secreting CgA and counteracting protein kinase C [49–51]. Reduced E-cadherin expression is not pronouncedly correlated with alcohol consumption, smoking status, familial history, or HP infection in this meta-analysis.

Some limitations deserve further attention in this study. Firstly, different antibody sources and dilutions bring bias into this meta-analysis. Secondly, there was heterogeneity in this study, as displayed in given tables and forest plots. A random-effects model was utilized to account for heterogeneity among studies. Subgroup analyses failed to clarify the source of heterogeneity. Thirdly, publication bias was present for differentiation grade. Fourthly, the inclusion of studies published in English may also introduce bias.

A conclusion can be drawn from this meta-analysis that the reduced expression of E-cadherin is significantly correlated with poor OS and unfavourable clinicopathological features in GC. The expression level of E-cadherin not only serves as a predictor for disease progression and prognosis in GC but also emerges as a novel therapeutic target.

## Supplementary Materials

Supplementary Figures
