# Supplementary material for: Reduced expression of E-cadherin correlates with poor prognosis and unfavorable clinicopathological features in gastric carcinoma: a meta-analysis
Source: Aging (Albany NY). 2024 Jun 12;16(12):10271–98. doi: 10.18632/aging.205929 (PMC11236327; doi:10.18632/aging.205929)
Supplement: Supplementary Figures [file aging-16-205929-s001.pdf]

# SUPPLEMENTARY FIGURES

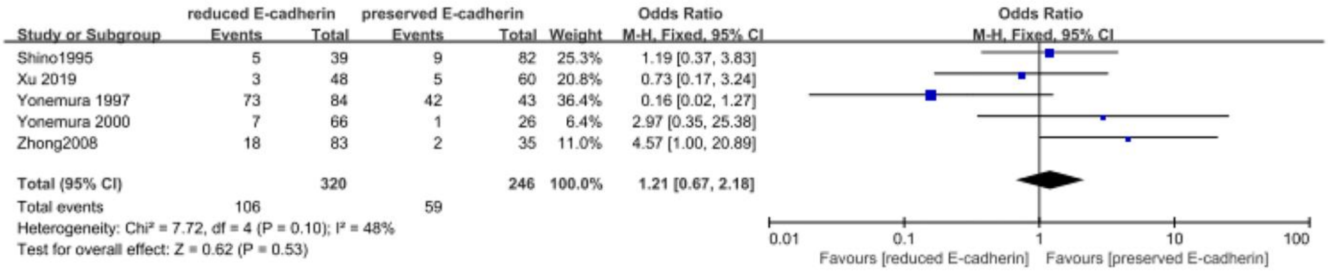


**Supplementary Figure 1. Forest plot of the odds ratio for the correlation of E-cadherin expression with liver metastasis.**

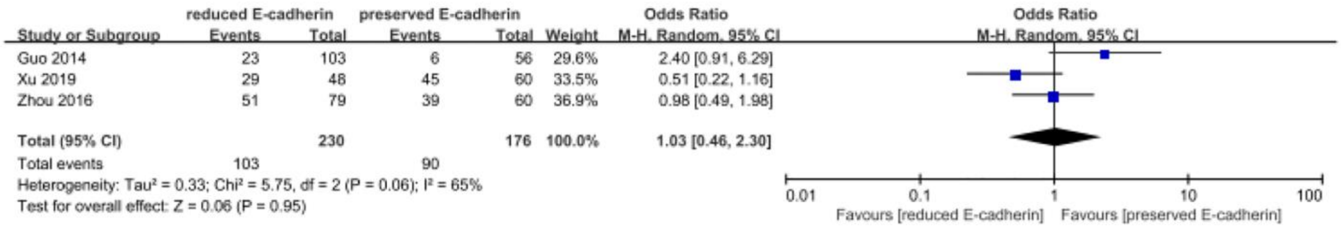


**Supplementary Figure 2. Forest plot of the odds ratio for the correlation of E-cadherin expression with perineural invasion.**

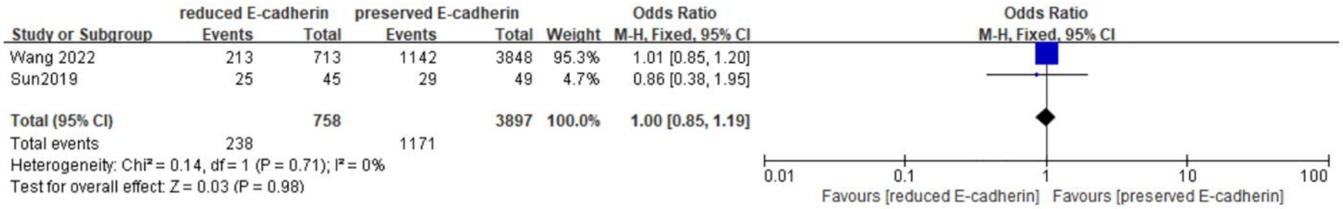


**Supplementary Figure 3. Forest plot of the odds ratio for the correlation of E-cadherin expression with alcohol consumption.**

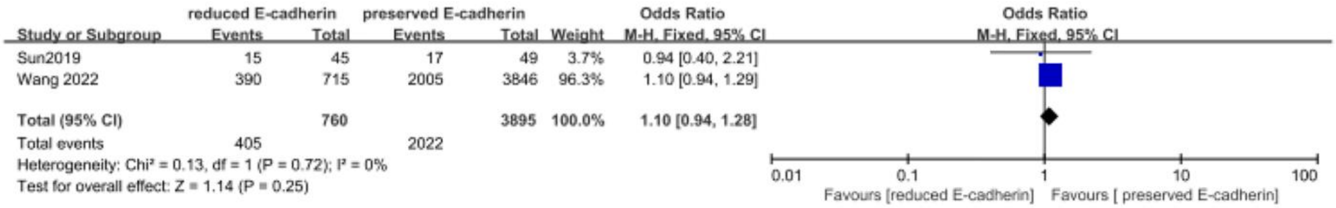


**Supplementary Figure 4. Forest plot of the odds ratio for the correlation of E-cadherin expression with smoking status.**

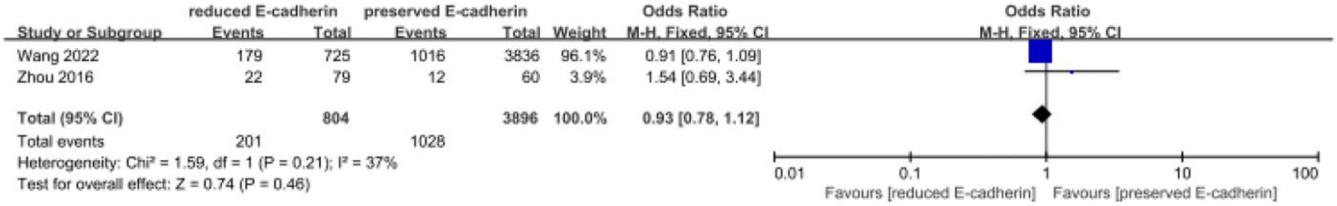


**Supplementary Figure 5. Forest plot of the odds ratio for the correlation of E-cadherin expression with familial history.**

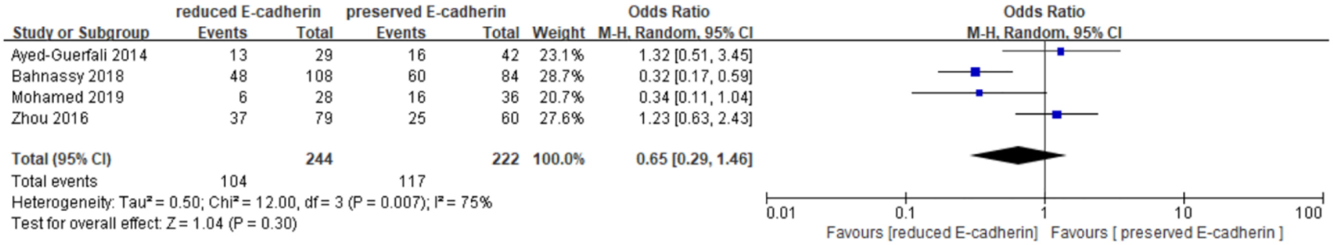


**Supplementary Figure 6. Forest plot of the odds ratio for the correlation of E-cadherin expression with HP infection.**

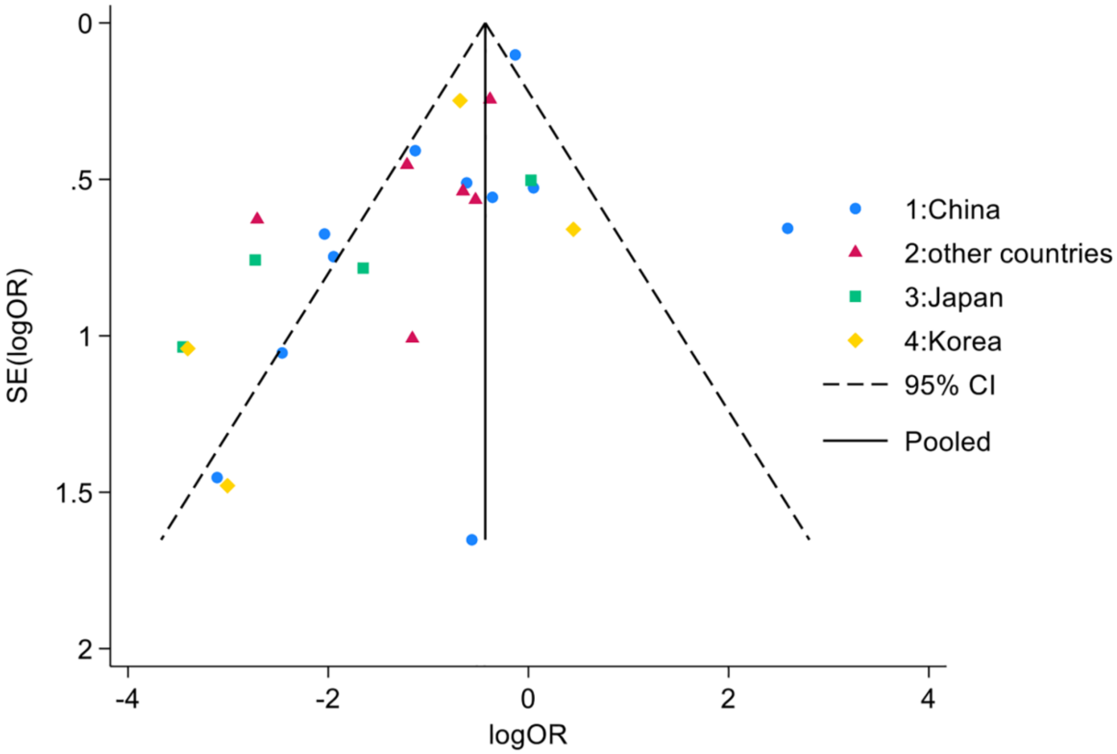


**Supplementary Figure 7. Funnel plot of the odds ratio for the correlation of E-cadherin expression with one-year overall survival.**

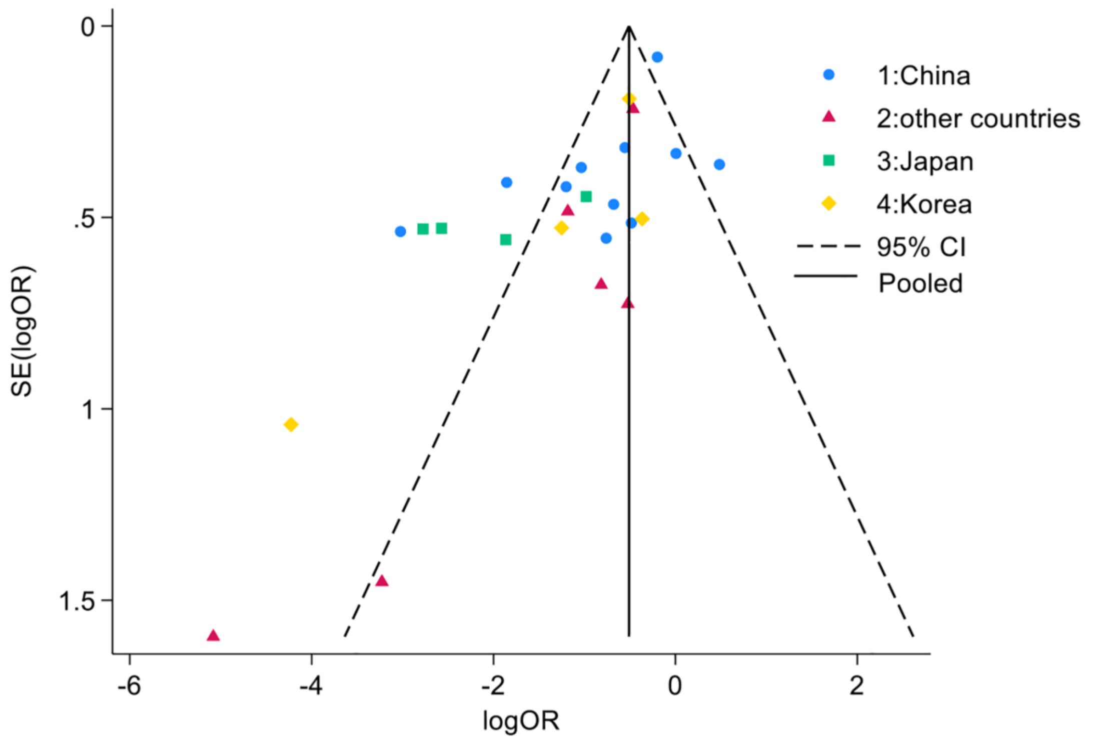


**Supplementary Figure 8. Funnel plot of the odds ratio for the correlation of E-cadherin expression with three-year overall survival.**

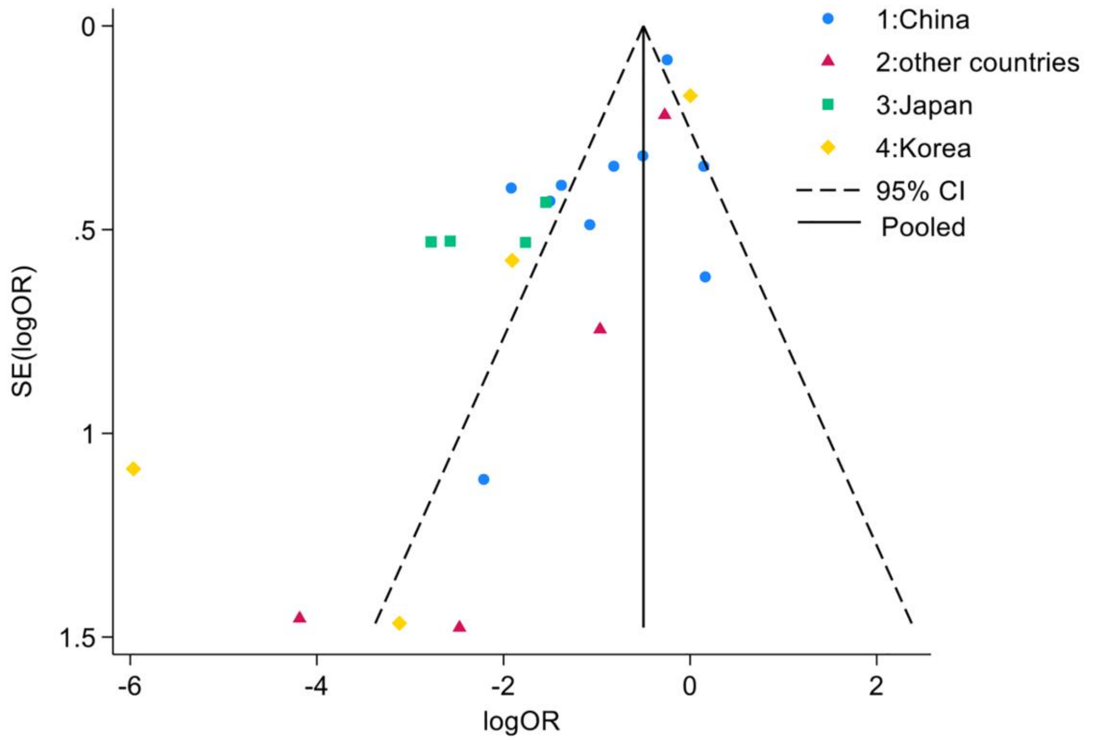


**Supplementary Figure 9. Funnel plot of the odds ratio for the correlation of E-cadherin expression with five-year overall survival.**

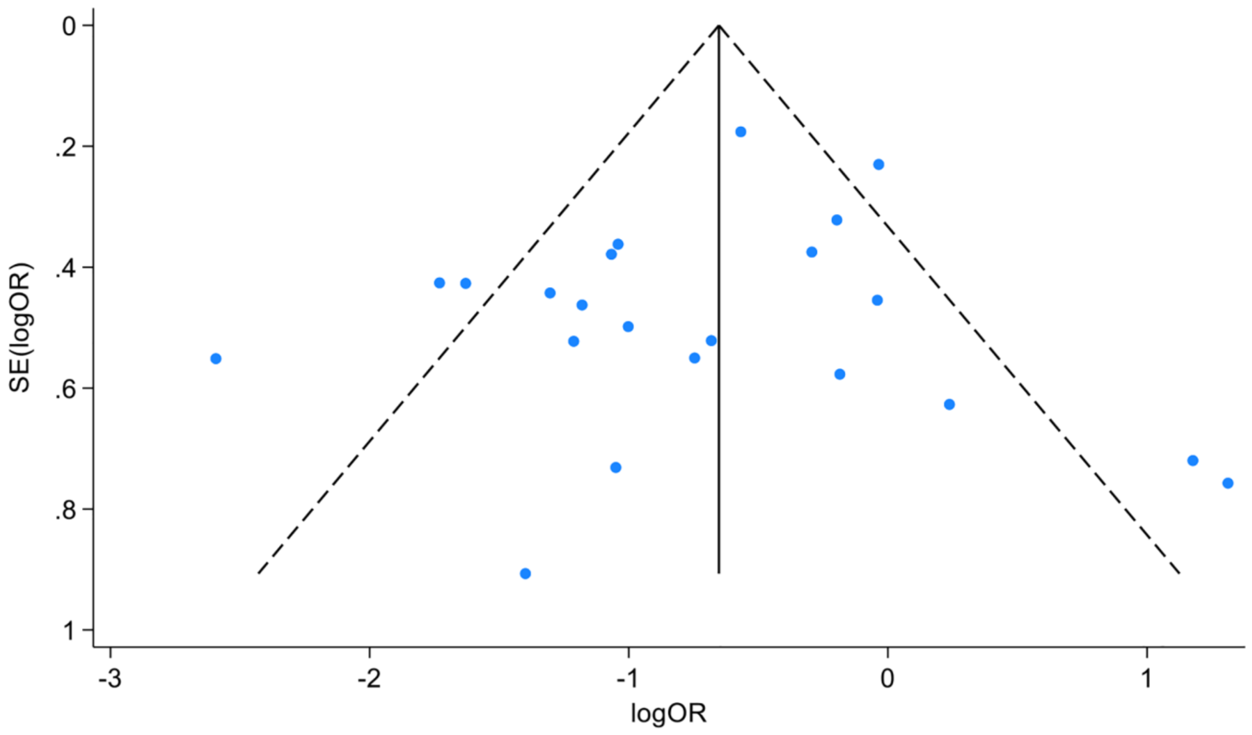


**Supplementary Figure 10. Funnel plot of the odds ratio for the correlation of E-cadherin expression with depth of invasion.**

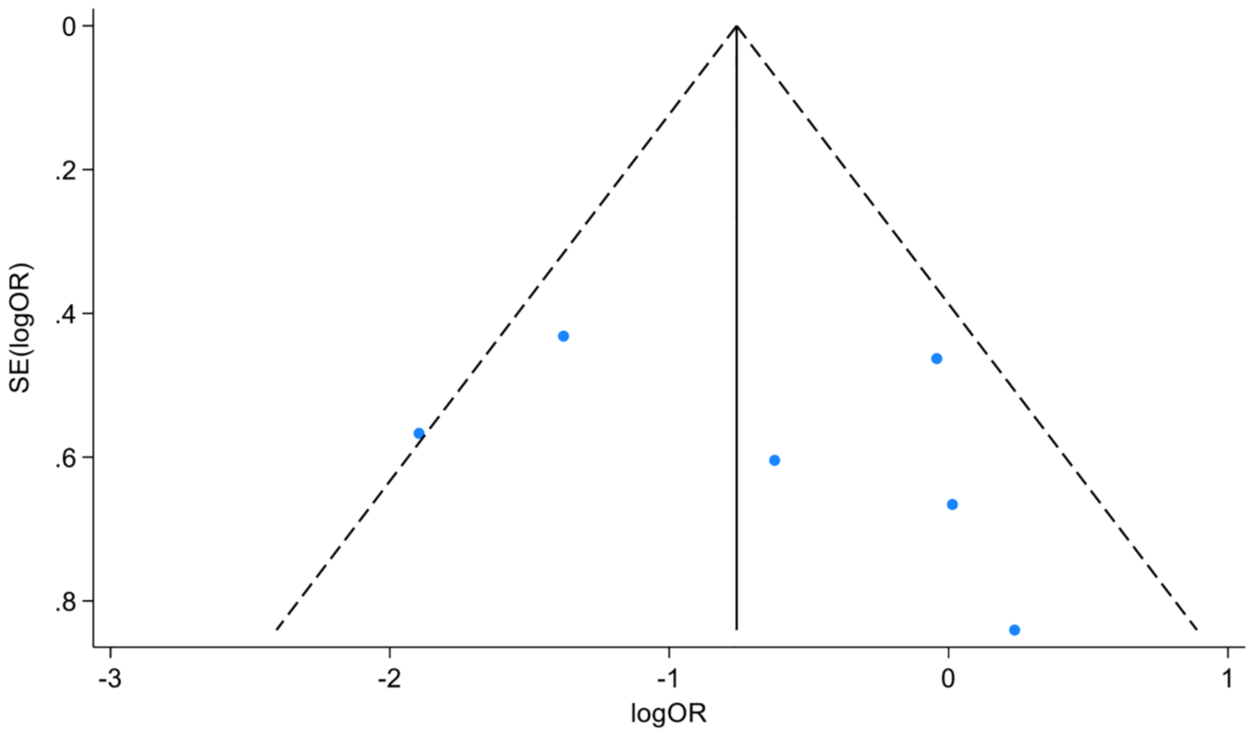


**Supplementary Figure 11. Funnel plot of the odds ratio for the correlation of E-cadherin expression with Borrmann classification.**

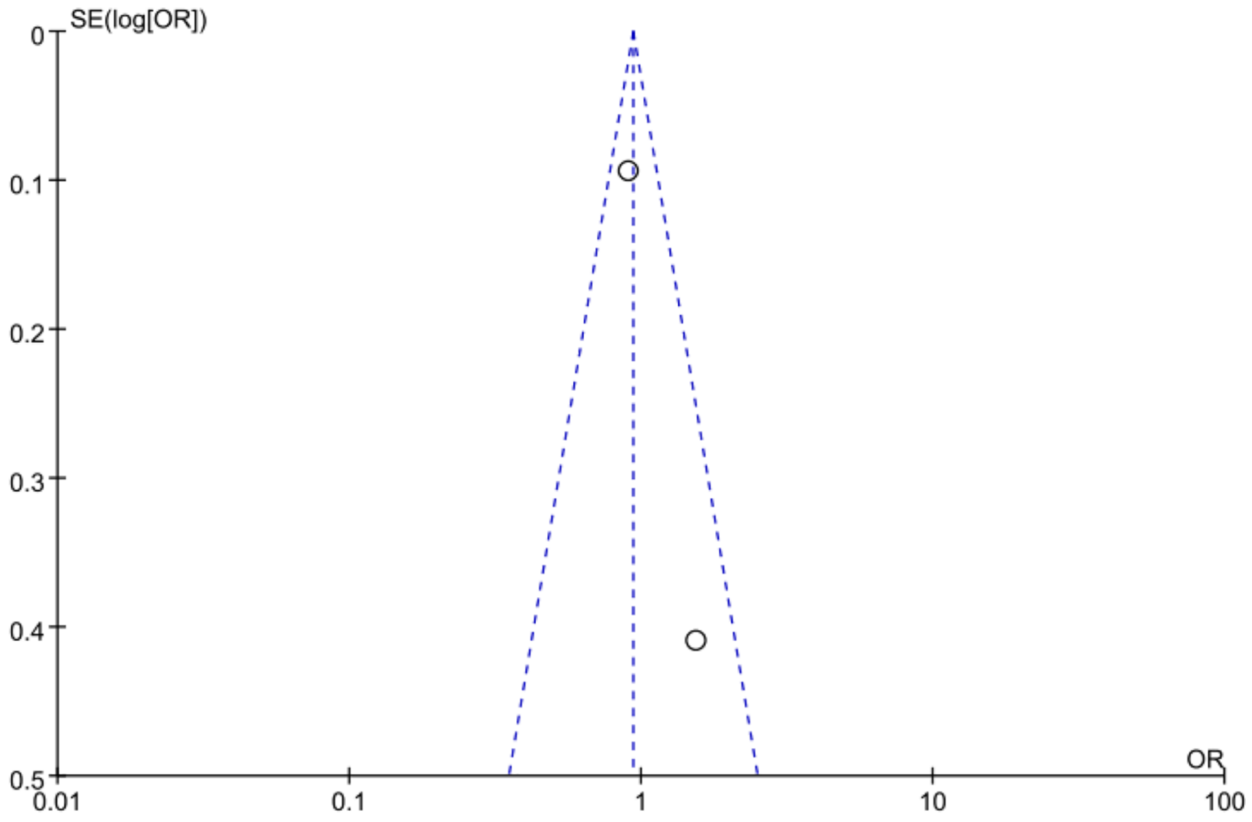


**Supplementary Figure 12. Funnel plot of the odds ratio for the correlation of E-cadherin expression with familial history.**

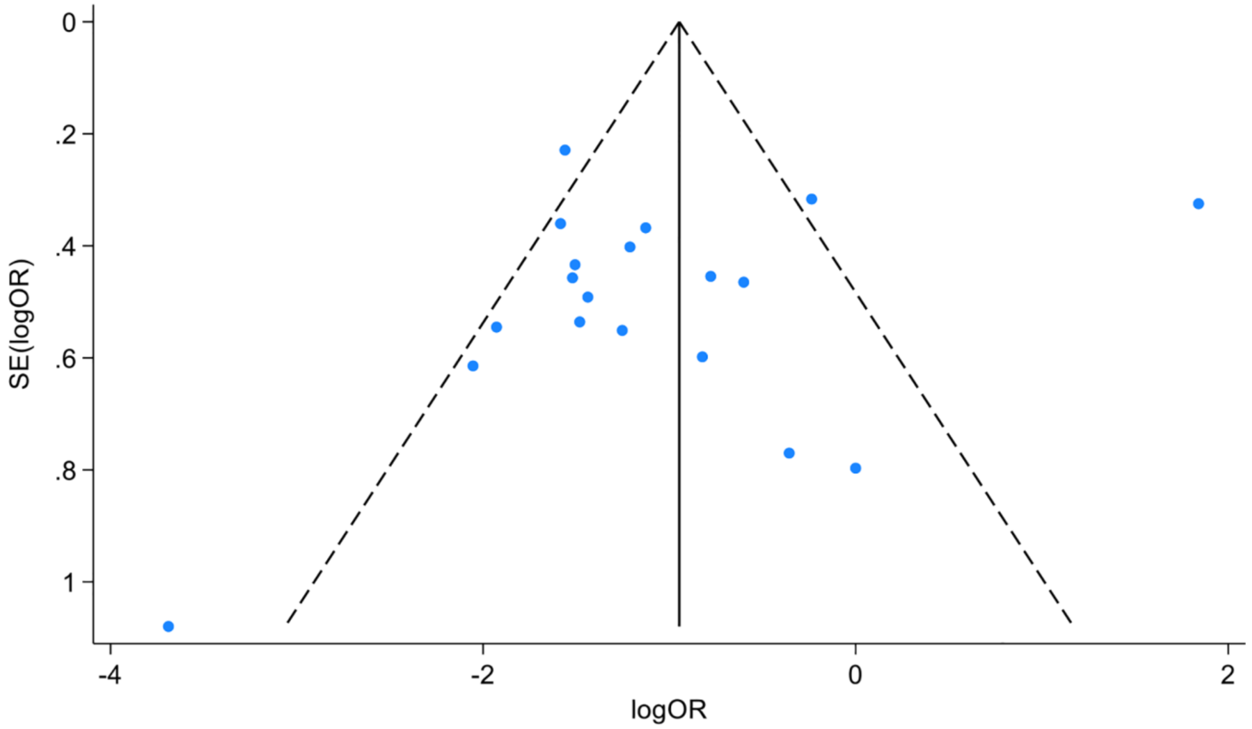


**Supplementary Figure 13. Funnel plot of the odds ratio for the correlation of E-cadherin expression with and Lauren type.**

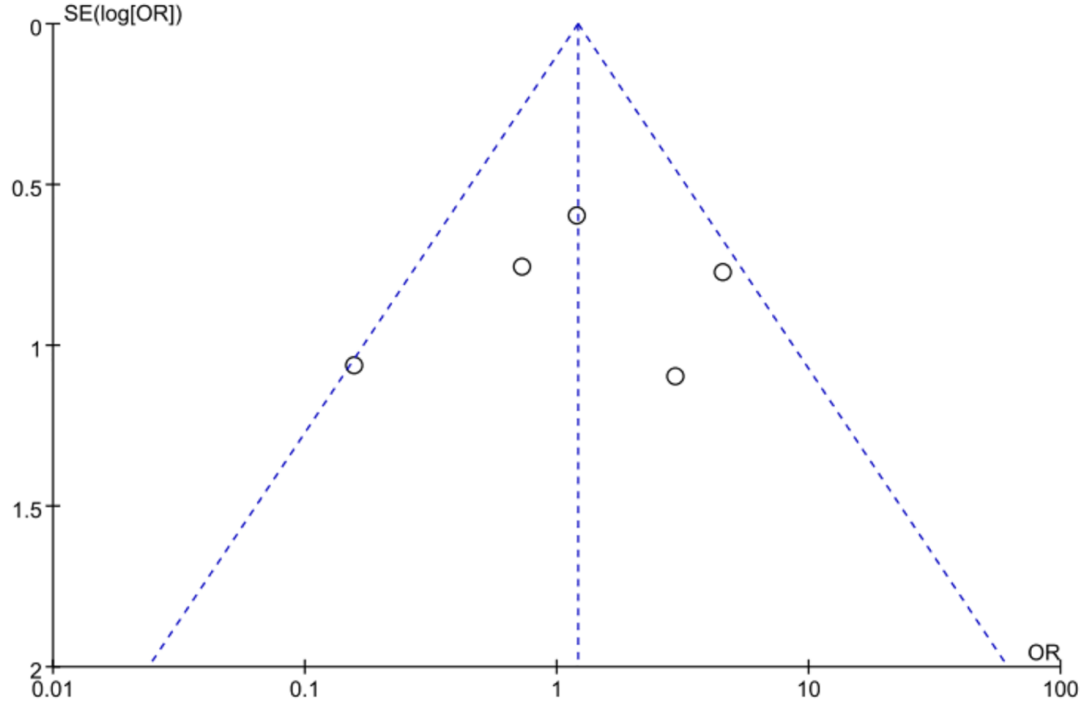


**Supplementary Figure 14. Funnel plot of the odds ratio for the correlation of E-cadherin expression with liver metastasis.**

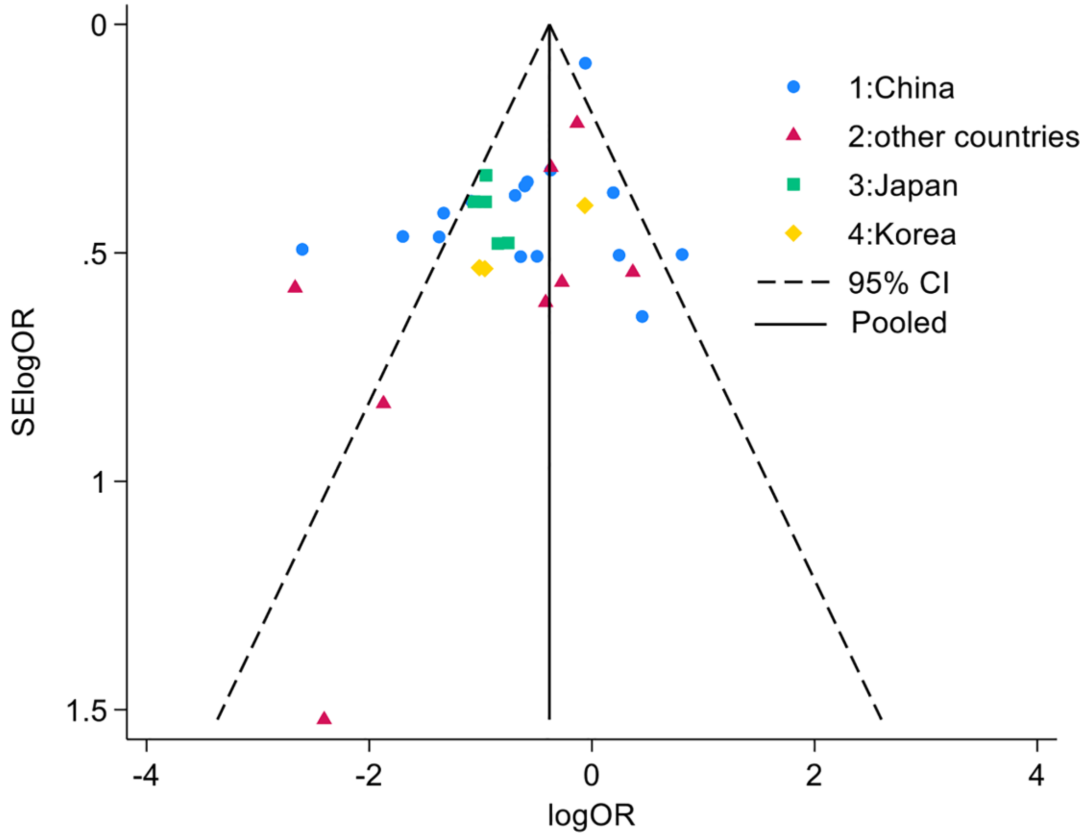


**Supplementary Figure 15. Funnel plot of the odds ratio for the correlation of E-cadherin expression with lymphatic node metastasis.**

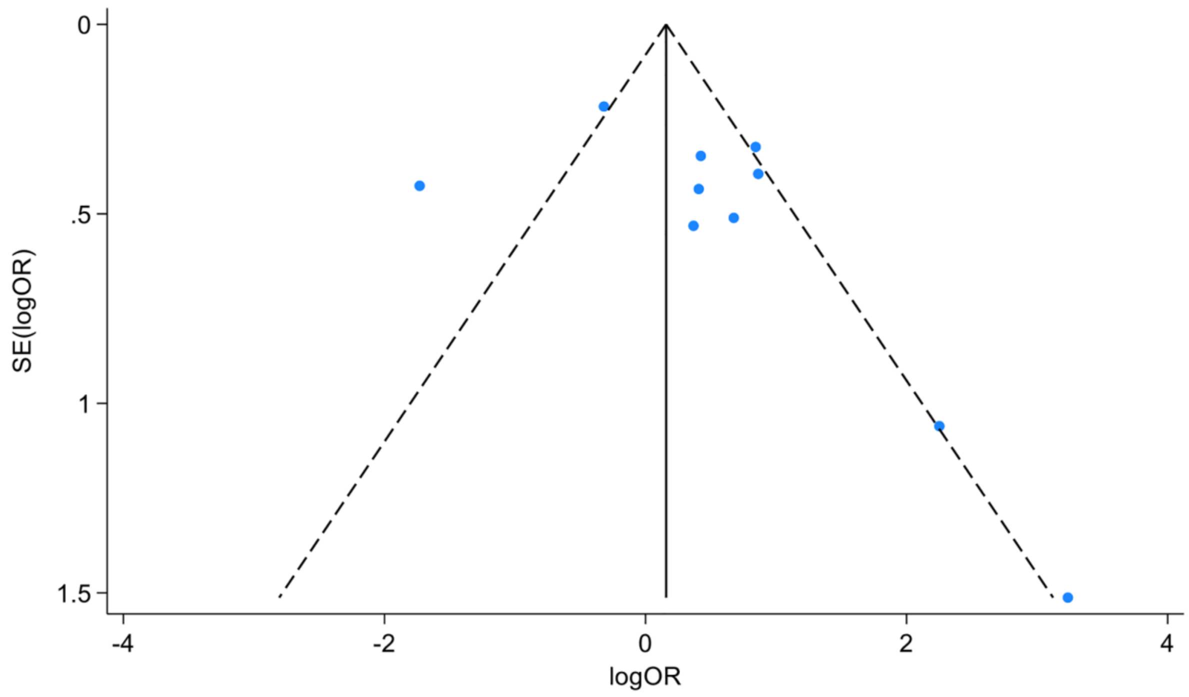


**Supplementary Figure 16. Funnel plot of the odds ratio for the correlation of E-cadherin expression with lymphatic vessel invasion.**

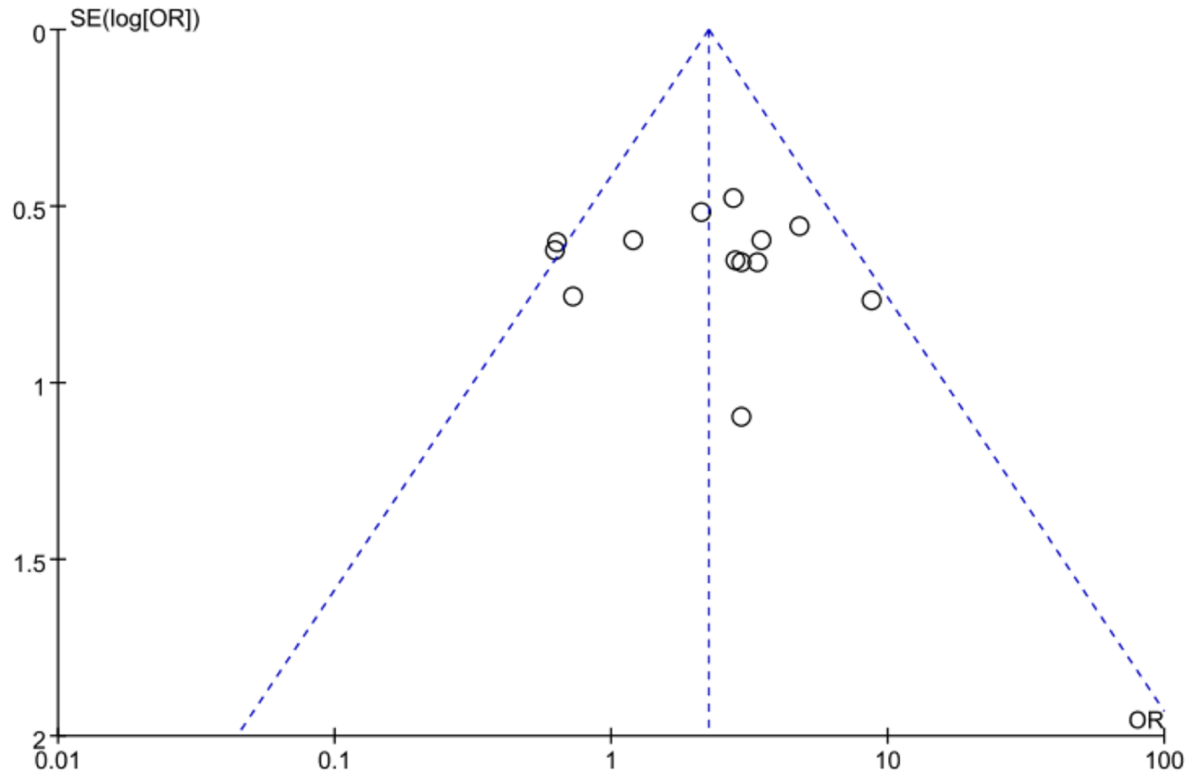


**Supplementary Figure 17. Funnel plot of the odds ratio for the correlation of E-cadherin expression with distant metastasis.**

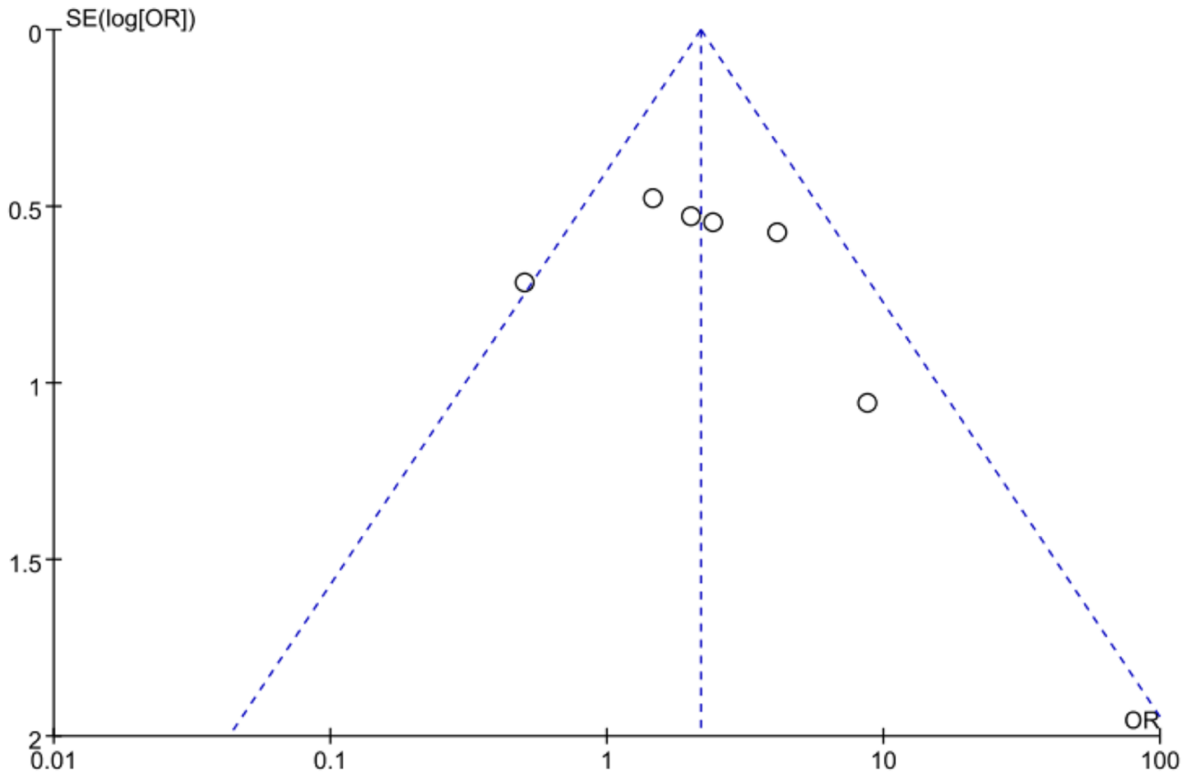


**Supplementary Figure 18. Funnel plot of the odds ratio for the correlation of E-cadherin expression with peritoneal metastasis.**

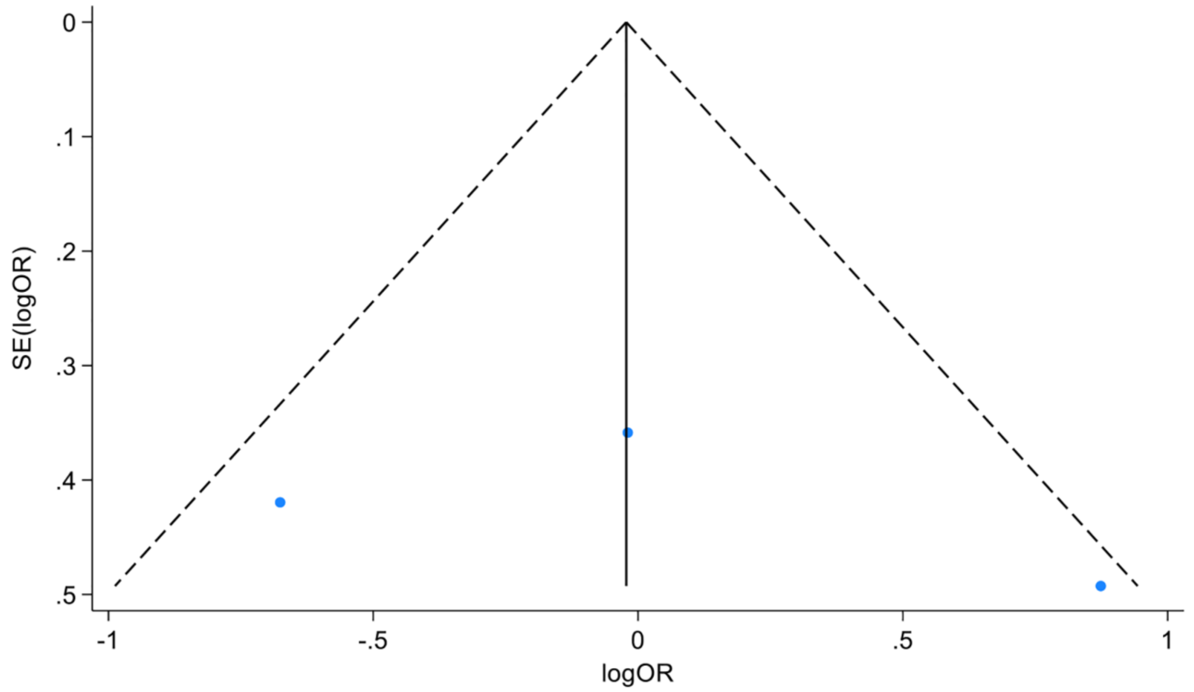


**Supplementary Figure 19. Funnel plot of the odds ratio for the correlation of E-cadherin expression with perineural invasion.**

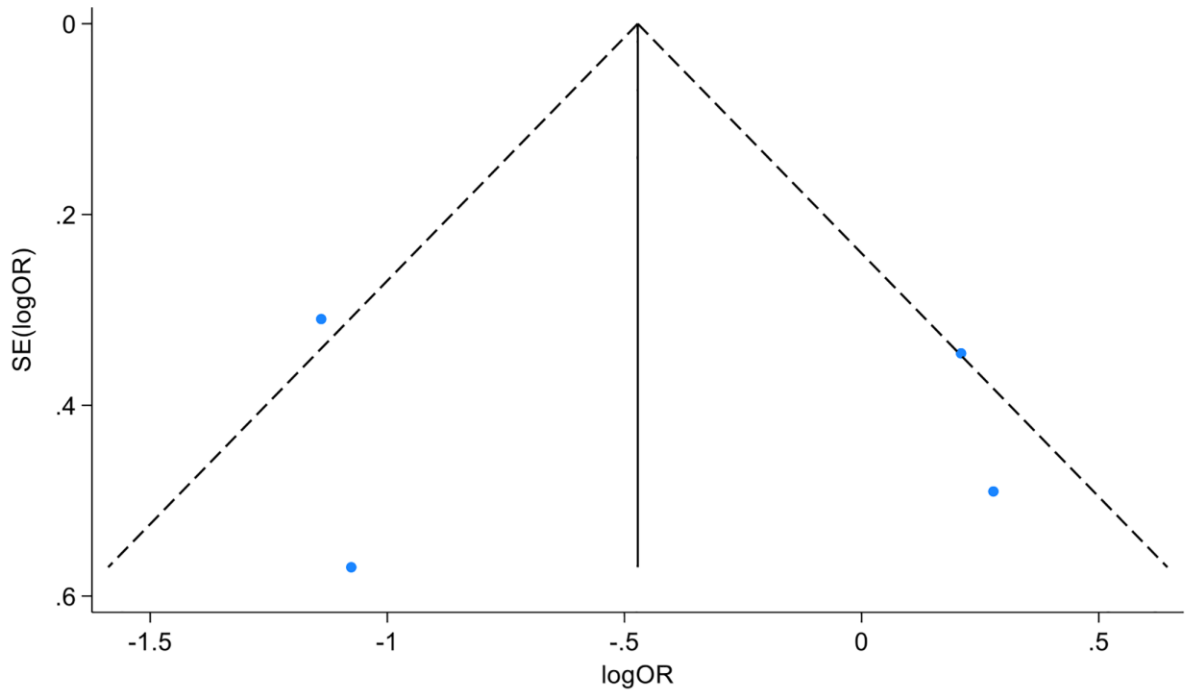


**Supplementary Figure 20. Funnel plot of the odds ratio for the correlation of E-cadherin expression with HP infection.**

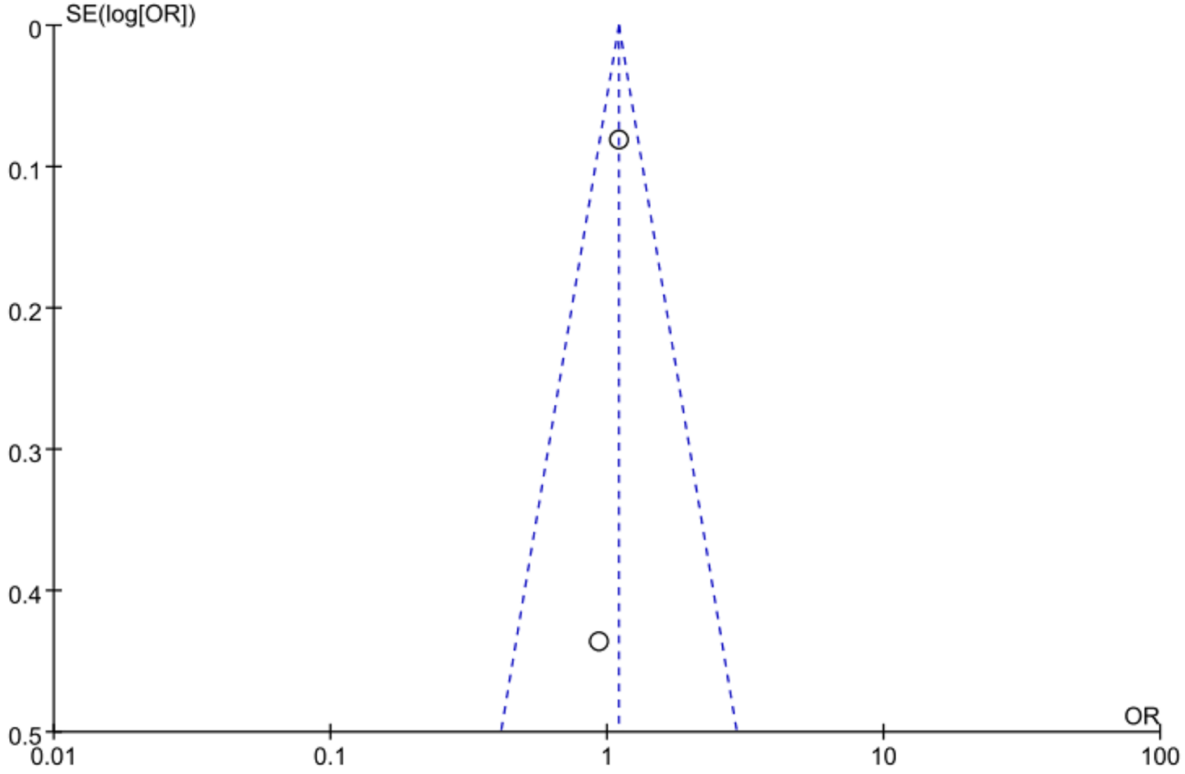


**Supplementary Figure 21. Funnel plot of the odds ratio for the correlation of E-cadherin expression with alcohol consumption.**

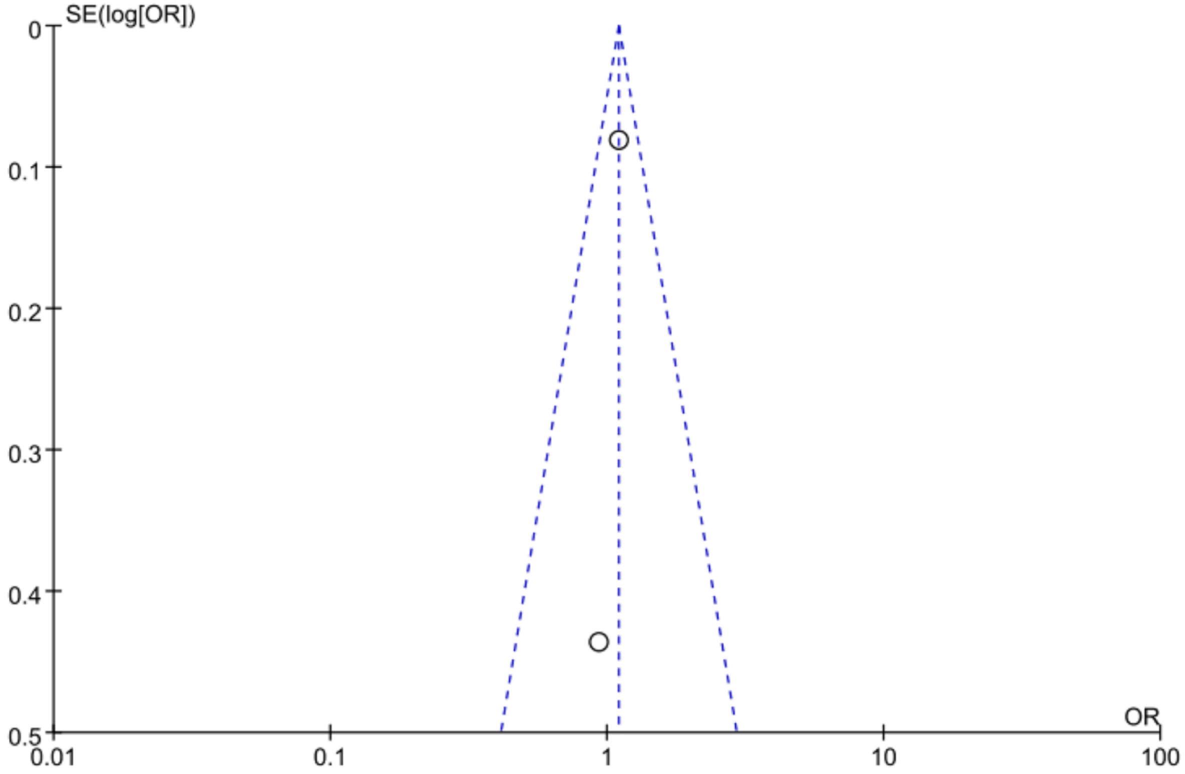


**Supplementary Figure 22. Funnel plot of the odds ratio for the correlation of E-cadherin expression with smoking status.**

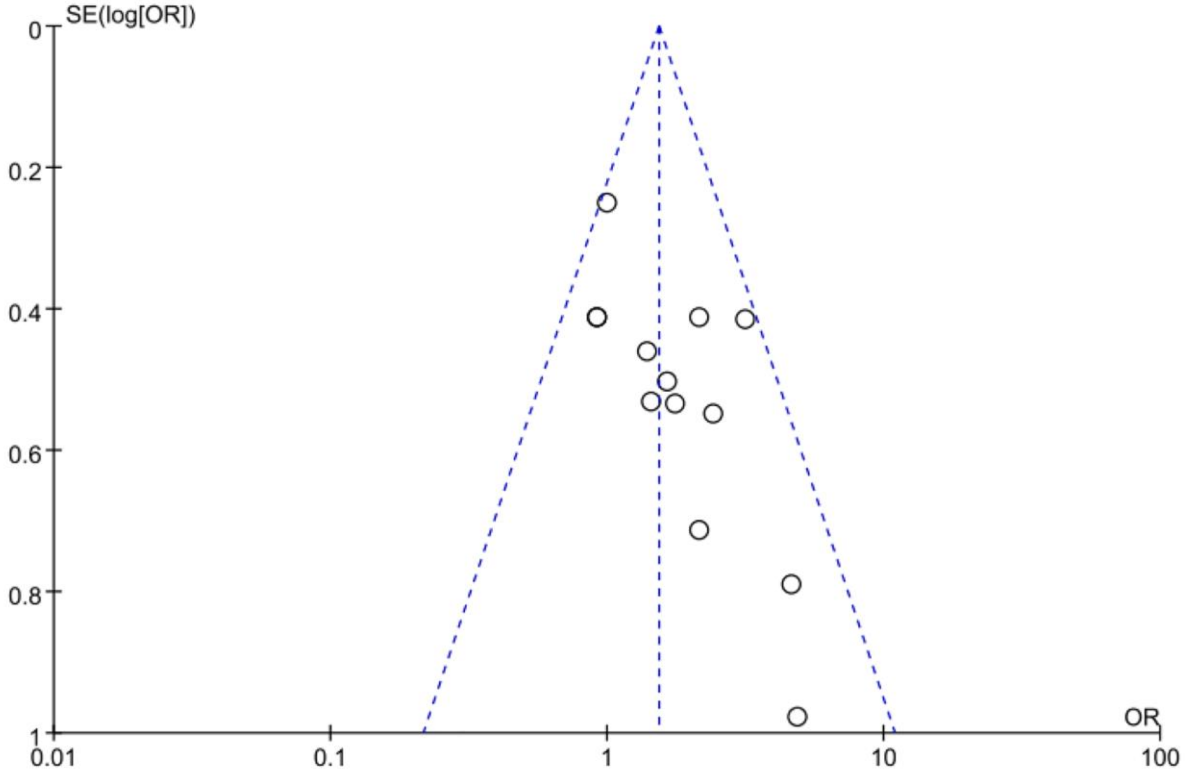


**Supplementary Figure 23. Funnel plot of the odds ratio for the correlation of E-cadherin expression with vascular invasion.**

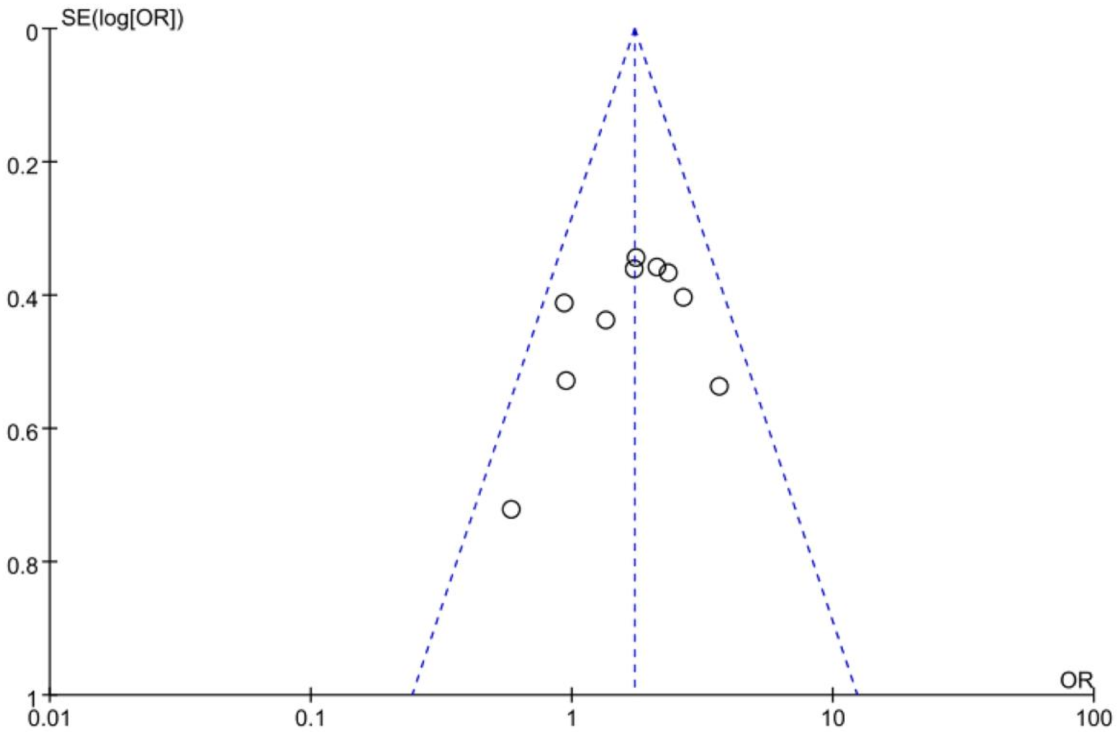


**Supplementary Figure 24. Funnel plot of the odds ratio for the correlation of E-cadherin expression with tumor size (≥5 cm vs. <5 cm).**

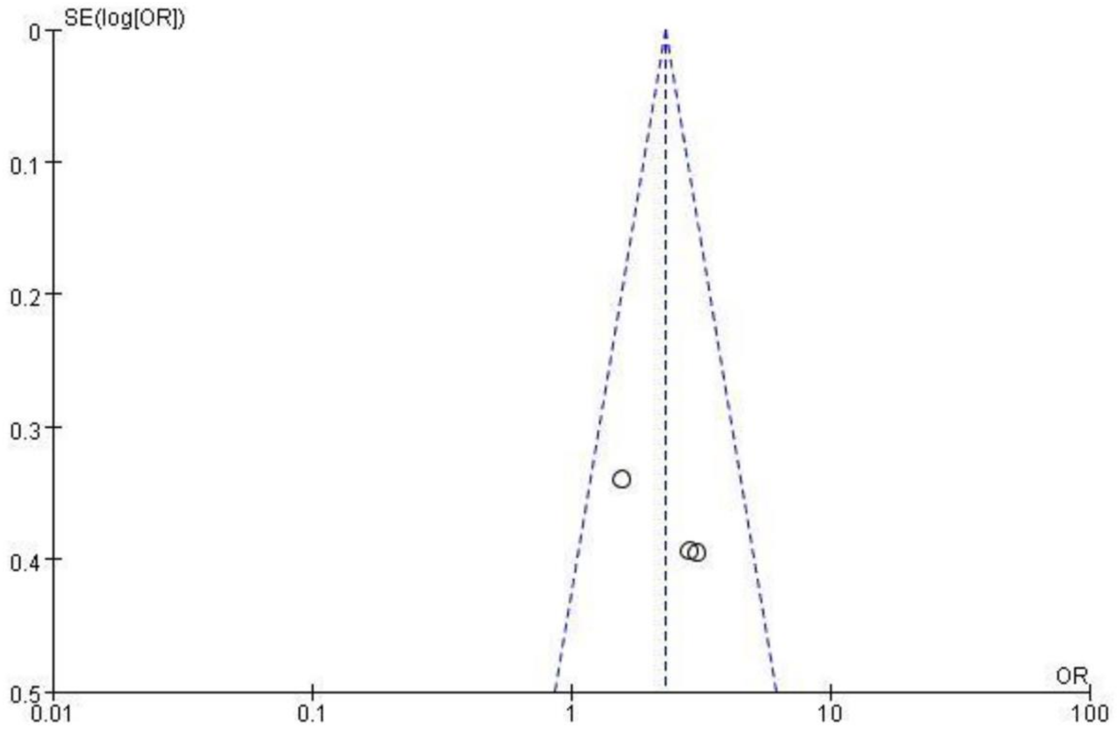


**Supplementary Figure 25. Funnel plot of the odds ratio for the correlation of E-cadherin expression with tumor size (≥6 cm vs. <6 cm).**

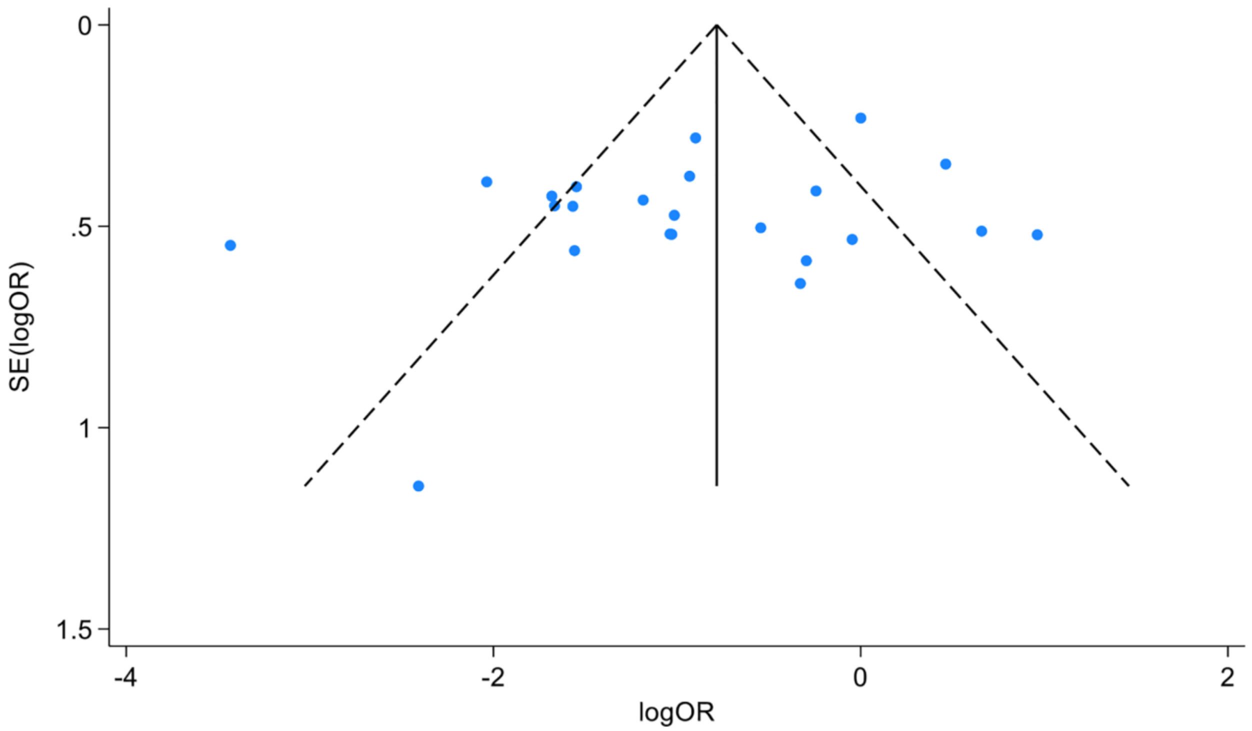


**Supplementary Figure 26. Funnel plot of the odds ratio for the correlation of E-cadherin expression with TNM stage.**

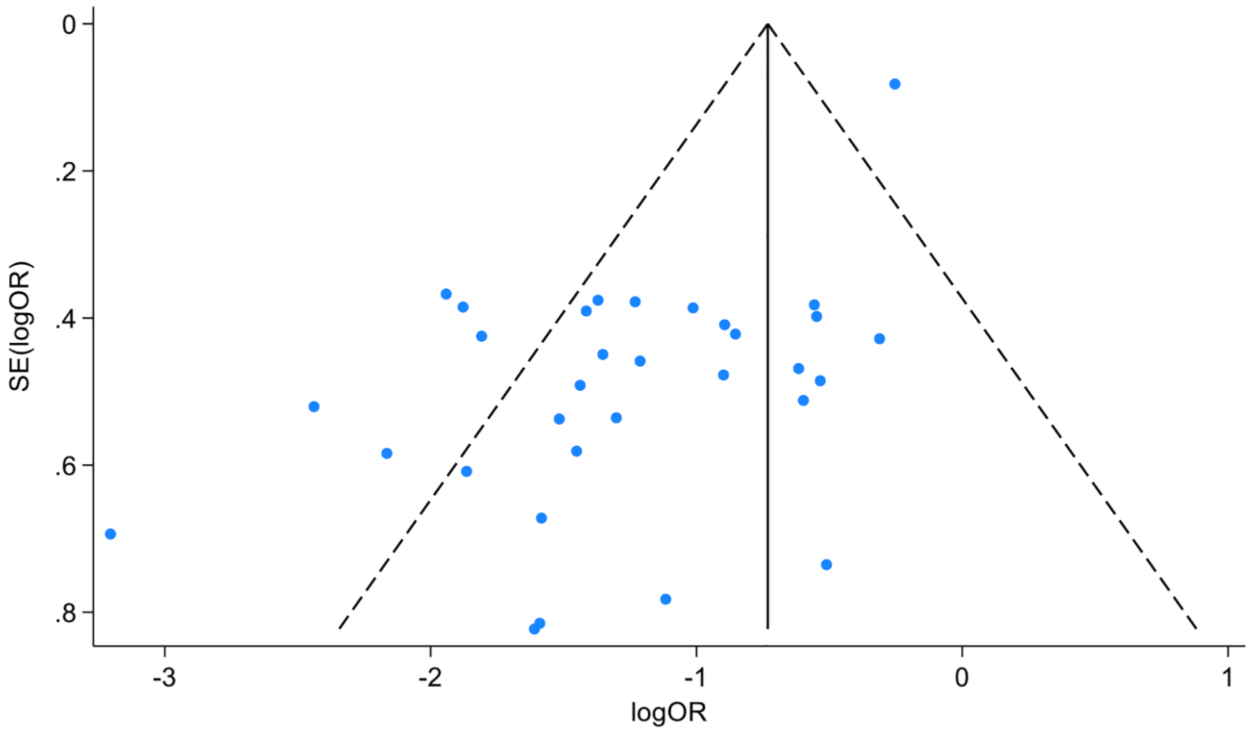


**Supplementary Figure 27. Funnel plot of the odds ratio for the correlation of E-cadherin expression with differentiation grade.**
